# BLIMP-1 and CEACAM1 cooperatively regulate human Treg homeostasis and function to control xenogeneic GVHD

**DOI:** 10.1172/jci.insight.183676

**Published:** 2025-08-07

**Authors:** Ying Ding, Aixin Yu, Milos Vujanac, Sabrina N. Copsel, Alejandro Moro, Luis Nivelo, Molly Dalzell, Nicolas Tchitchek, Michelle Rosenzwajg, Alejandro V. Villarino, Robert B. Levy, David Klatzmann, Thomas R. Malek

**Affiliations:** 1 Department of Microbiology and Immunology, Miller School of Medicine, University of Miami, Miami, Florida, USA.; 2 Sorbonne Université, UPMC Univ Paris 06, UMRS 959, Immunology-Immunopathology-Immunotherapy (I3), Paris, France.

**Keywords:** Autoimmunity, Immunology, Adaptive immunity, Autoimmune diseases, Immunotherapy

## Abstract

Regulatory T cells (Tregs) are essential for peripheral tolerance and depend on TCR and IL-2 receptor (IL-2R) signaling for their homeostasis and function. In mice, IL-2–dependent B-lymphocyte-induced maturation protein 1 (BLIMP-1) contributes to Treg homeostasis. BLIMP-1 is a major transcriptional hub in human Tregs, but its mechanisms of action remain undefined. Here, using CRISPR/Cas9 ablation, we show that BLIMP-1 limits human Treg proliferation but supports IL-10, cytotoxic T lymphocyte-associated protein 4, several immune checkpoints including carcinoembryonic antigen-related cell adhesion molecule 1 (CEACAM1), and Treg functional activity. BLIMP-1 restrains Treg expansion to IL-2 by downregulating CD25 and IL-2R signaling, and by enhancing CEACAM1 expression, which in turn inhibits responsiveness to CD3/CD28 signaling and activation of mTOR. Prolonged IL-2R signaling optimizes BLIMP-1 expression, supporting chromosomal opening of *CEACAM1* to increased CEACAM1 expression through STAT5- and BLIMP-1–driven enhancers. Correspondingly, CEACAM1 is highly induced on Tregs from patients with autoimmune disease undergoing low-dose IL-2 therapy, and these Tregs showed reduced proliferation. A humanized mouse model of xenogeneic graft-versus-host disease demonstrates that BLIMP-1 normally promotes, while CEACAM1 restrains, Treg suppressive activity. Collectively, our findings reveal that BLIMP-1 and CEACAM1 function in an IL-2–dependent feedback loop to restrain Treg proliferation and affect suppressive function. CEACAM1 also acts as a highly selective biomarker of IL-2R signaling in human T cells.

## Abstract 2

BLIMP-1 and CEACAM1 form a negative regulatory loop that limits human Treg expansion while regulating their function. This regulatory loop is active in Treg-dependent control of xenogeneic graft versus host disease.

## Graphical abstract

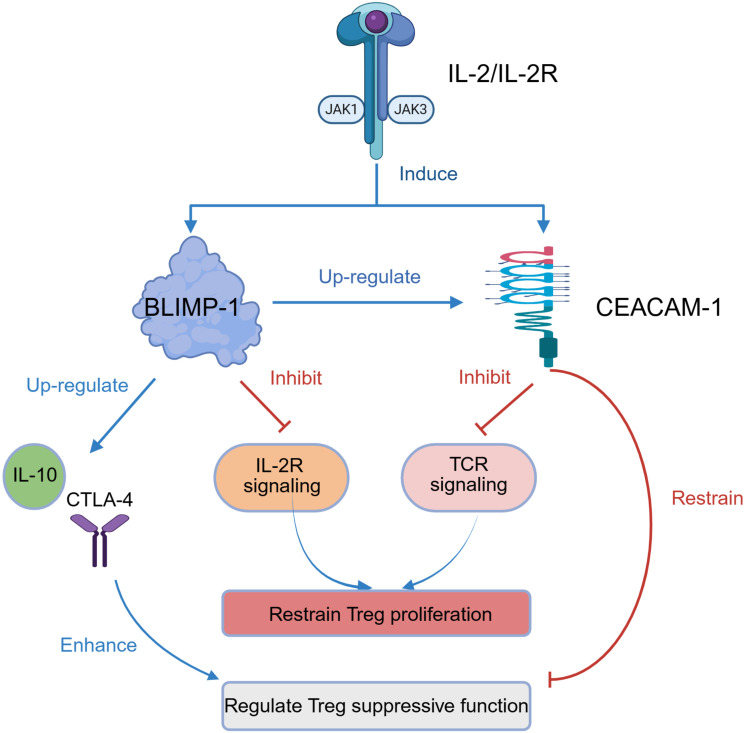

## Introduction

The homeostasis and function of regulatory T cells (Tregs) rely on coordinated TCR and IL-2 receptor (IL-2R) signaling. In peripheral immune compartments, physiological IL-2 levels support IL-2R signaling that is essential for Treg survival (1) whereas TCR signaling drives Treg activation and development into highly suppressive effector Tregs capable of migrating into inflamed sites (2). Low-dose IL-2 therapies in autoimmune and other inflammatory diseases, including chronic graft-versus-host disease (GVHD), promote Treg expansion resulting in clinical improvement (3, 4). The mechanisms by which IL-2R and TCR signaling regulate the Treg compartment are not fully defined but are relevant to understanding the normal physiological processes by which Tregs maintain peripheral tolerance and therapeutic responses to low-dose IL-2. One downstream target of IL-2R signaling is B-lymphocyte-induced maturation protein 1 (BLIMP-1) (1, 5–7), which acts in Tregs as well as CD4^+^ and CD8^+^ T effector (Teff) cells. Another potential target is carcinoembryonic antigen-related cell adhesion molecule 1 (CEACAM1), a known inhibitor of TCR signaling in Teff cells (8, 9), whose function in Tregs remains largely unexplored.

BLIMP-1, encoded by *PRDM1*, is a broadly expressed transcriptional regulator with diverse functions in T cells. BLIMP-1 suppresses activated Teff cell proliferation and increases susceptibility to activation-induced cell death (10, 11). In CD4^+^ Teff cells, BLIMP-1 promotes T helper 2 (Th2) cells while limiting Th1, T follicular helper, and Th17 cell development (12, 13). In CD8^+^ T cells, BLIMP-1 is essential for terminal differentiation of cytotoxic T lymphocytes (CTLs) (14) and supports T cell exhaustion (15). In Tregs, BLIMP-1 is highly expressed in activated effector Tregs and cooperates with interferon regulatory factor 4 (IRF4) for IL-10 production (16, 17). T cell–specific BLIMP-1 deficiency leads to inflammatory bowel disease and aggravates experimental autoimmune encephalomyelitis (18, 19). However, BLIMP-1–deficient Tregs are suppressive in vitro and protect lymphopenic hosts from colitis, suggesting a complex and context-dependent role in Treg function (10).

CEACAM1, a type 1 glycoprotein with multiple isoforms and broad tissue distribution, is another molecule that regulates Teff cells. CEACAM1 is upregulated in CD4^+^ and CD8^+^ Teff cells following TCR and IL-2R signaling, with the long isoform predominating (20–22). This isoform contains 2 immunoreceptor tyrosine based inhibitory motifs (ITIMs) that limit TCR signaling to reduce proliferation, Th1 cytokine secretion, and CTL activity (23). Thus, BLIMP-1, at the transcriptional level, and CEACAM1, at proximal TCR signaling, function to regulate the proliferation and function of Teff cells.

Most insights into BLIMP-1 function in T cells, including Tregs, come from mouse models (24, 25). With respect to human Tregs, a recent study identified BLIMP-1 as a FOXP3-independent transcriptional node controlling distinct gene networks (26). However, its potential function and mechanism of action in human Tregs remain unclear. Moreover, the expression, regulation, and function of CEACAM1 in Tregs have not been explored. In this study, we use CRISPR/Cas9 to knock out BLIMP-1α and CEACAM1 in human Tregs, revealing that both are IL-2R dependent and act in concert in a feedback loop to limit proliferation to IL-2– and CD3/CD28-induced signaling, respectively. The functional relevance of this loop is explored in a chronic xenogeneic GVHD (xGVHD) model and in patients with autoimmune disease undergoing low-dose IL-2 therapy. These findings provide insights into human Treg homeostasis and function.

## Results

### Efficient knockout of PRDM1 in human Tregs using CRISPR/Cas9.

**Figure 1:**
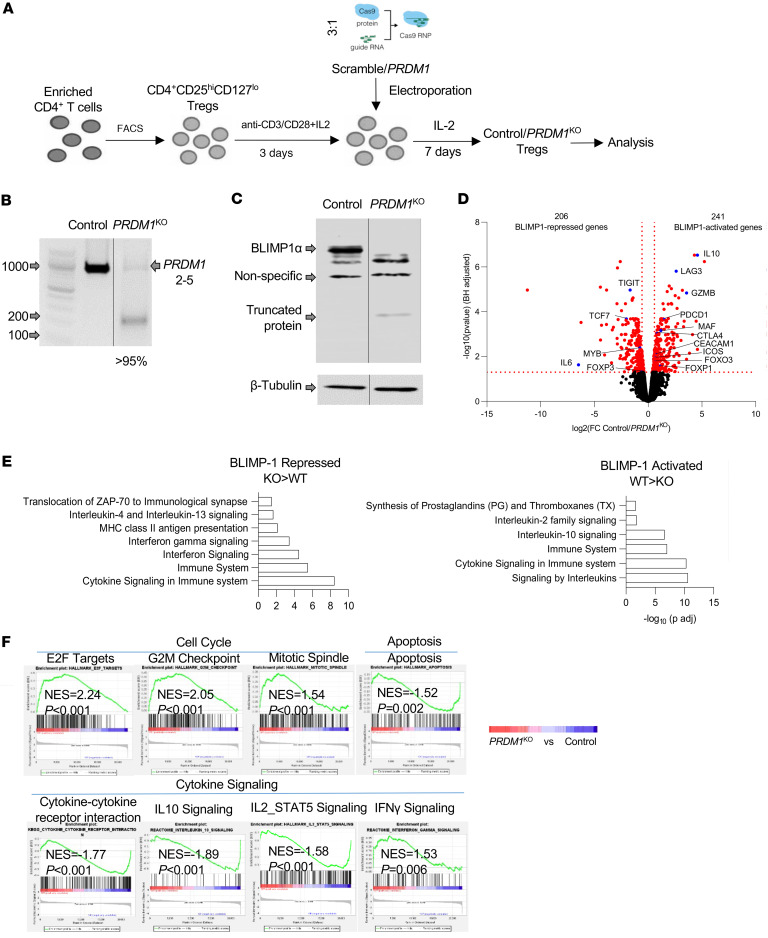
BLIMP-1–dependent transcriptional programs in human Tregs. (**A**) Schematic of culture conditions and Cas9:single-guide RNA ribonucleoprotein (Cas9 RNP) delivery. (**B**) BLIMP-1 mRNA expression in control and *PRDM1*^KO^ Tregs was measured by RT-PCR. Primers were designed to amplify a 910 bp region spanning exon 2 (upstream of the sgRNA site) to exon 5 (downstream of the editing site). Knockout efficiency is shown. Units are bp. (**C**) Immunoblot analysis of BLIMP-1 protein. The black vertical lines on **B** and **C** indicate that lanes were run on the same gel or blot but were noncontiguous. (**D**–**F**) At 7 days posttransfection, Tregs were rested overnight in media and stimulated with anti-CD3/CD28 and IL-2 for 16 hours before RNA-Seq. (**D**) Volcano plot comparing gene expression in control and *PRDM1*^KO^ Tregs. Genes with ≥1.5-fold change (FC) and FDR < 0.05 are colored in red. (**E**) Reactome pathway analysis of DEGs. (**F**) Representative gene set enrichment analysis (GSEA) plots illustrating transcriptional signatures in *PRDM1*^KO^ versus control Tregs, using gene sets from Molecular Signatures Database (MSigDB) (Hallmark, KEGG, and Reactome). Normalized enrichment score (NES) and *P* value are indicated.

To study the function of BLIMP-1, *PRDM1* was ablated by CRISPR/Cas9, which yields high editing efficiency of primary T cells (26, 27), during the culture of purified Tregs stimulated with anti-CD3/CD28 and IL-2 (Figure 1A). Two sgRNAs targeting coding exon 2 and exon 5 of *PRDM1* were selected based on predicted on-target efficiency (Supplemental Figure 1A; supplemental material available online with this article; https://doi.org/10.1172/jci.insight.183676DS1), as these sgRNAs are at upstream DNA sequences coding for 2 essential protein domains for BLIMP-1 function, the regulatory PR/SET domain and the DNA-binding zinc finger domain. High knockout efficiency of *PRDM1* was confirmed (>90%) at the DNA and RNA levels by the T7 endonuclease I (T7EI) assay (Supplemental Figure 1B), cDNA sequencing surrounding exons 2 and 5 (Supplemental Figure 1C), and reverse transcription PCR (RT-PCR) (Figure 1B). BLIMP-1α, the full-length, fully functional, and dominantly expressed isoform of BLIMP-1, was undetectable by Western blotting (Figure 1C).

### BLIMP-1–dependent transcriptional programs in human Tregs.

To gain an unbiased insight into the contribution of BLIMP-1 in human Tregs, we determined the transcriptional profile of control scrambled and *PRDM1*-deleted Tregs. At 7 days after transfection of the sgRNAs and purified Cas9 followed by expansion in IL-2, Tregs were rested overnight and then restimulated through the TCR, CD28, and IL-2R for 16 hours prior to isolation of RNA for genome-wide RNA-Seq. We identified 447 transcripts as differentially expressed genes (DEGs; >1.5-fold, FDR < 0.05). Volcano plots show 206 BLIMP-1–repressed (genes with enhanced expression upon deletion) and 241 BLIMP-1–activated (genes with diminished expression upon deletion) DEGs (Figure 1D). Many DEGs were identified with established functions in T cells. BLIMP-1–activated genes included those implicated in Treg function (*IL-10*, *GZMB*), immune checkpoints (*PDCD1*, *CTLA4*, *LAG3*, *CEACAM1*, and *ICOS*), and transcriptional regulators (*MAF*, *FOXO3*, and *FOXP1*). BLIMP-1–repressed genes included *TIGIT*, *IL6*, and transcription factors (*FOXP3*, *TCF7*, and *MYB*).

Hypergeometric testing (HGT) of BLIMP-1–regulated gene sets against the Reactome (Figure 1E) and Kyoto Encyclopedia of Genes and Genomes (KEGG) (Supplemental Figure 2) pathway databases indicated that BLIMP-1influences numerous immune processes. Consistent with previous studies (17), BLIMP-1 positively contributes to the IL-10 signaling pathway (Figure 1E). Genes in this enrichment group include *IL10*, *CCL4*, *CCL3*, *CCR5*, *CCR2*, *FCER2*, *IL1RN*, *CCL3L1*, *IL1A*, and *PTGS2*, suggesting that BLIMP-1 functions to coordinate production of not only IL-10 but also other cytokines and chemokines in human Tregs. Genes negatively regulated by BLIMP-1 are enriched in the IFN and IFN-γ signaling pathways (Figure 1E). Many genes (*CIITA*, *HLA-DRA*, *TRIM22*, *IFITM1*, *HLA-DPA1*, *IFNGR2*, *STAT1*, *IFITM2*, *IFITM3*, *HLA-DPB1*, *TRIM5*, *HLA-DRB1*, *IFIT2*, *IRF8*) in these enrichment groups are either directly involved in the response to IFNs or are IFN-responsive genes, suggesting that BLIMP-1 functions to limit the response of human Tregs to IFNs, which might promote their stability. Also notable, Reactome identified IL-2 family signaling as a BLIMP-1–dependent process (Figure 1E). The genes involved in the IL-2 family cytokine signaling activated by BLIMP-1 are *IL2RB*, *IL3RA*, *IL5*, *IL9*, *IL9R*, *HAVCR2*, *IL4*, *IL10*, *IL13*, and *PDGFRB*. This enrichment largely reflects cytokines and their receptors rather than components of the signaling mechanism. Comparable results were obtained using gene set enrichment analysis (GSEA), which, additionally, suggests a role for BLIMP-1 in limiting cell cycle progression (Figure 1F). GSEA also suggests a role for BLIMP-1 in regulation of IL-2/STAT5 signaling. Collectively, these findings are consistent with the role for BLIMP-1 in limiting human Treg proliferation and responsiveness to IFNs while promoting their functional activity by secretion of IL-10 and other cytokines/chemokines. Moreover, IL-2/STAT5 signaling appears to be both positively and negatively regulated by BLIMP-1.

### BLIMP-1 limits proliferation of human Tregs.

**Figure 2:**
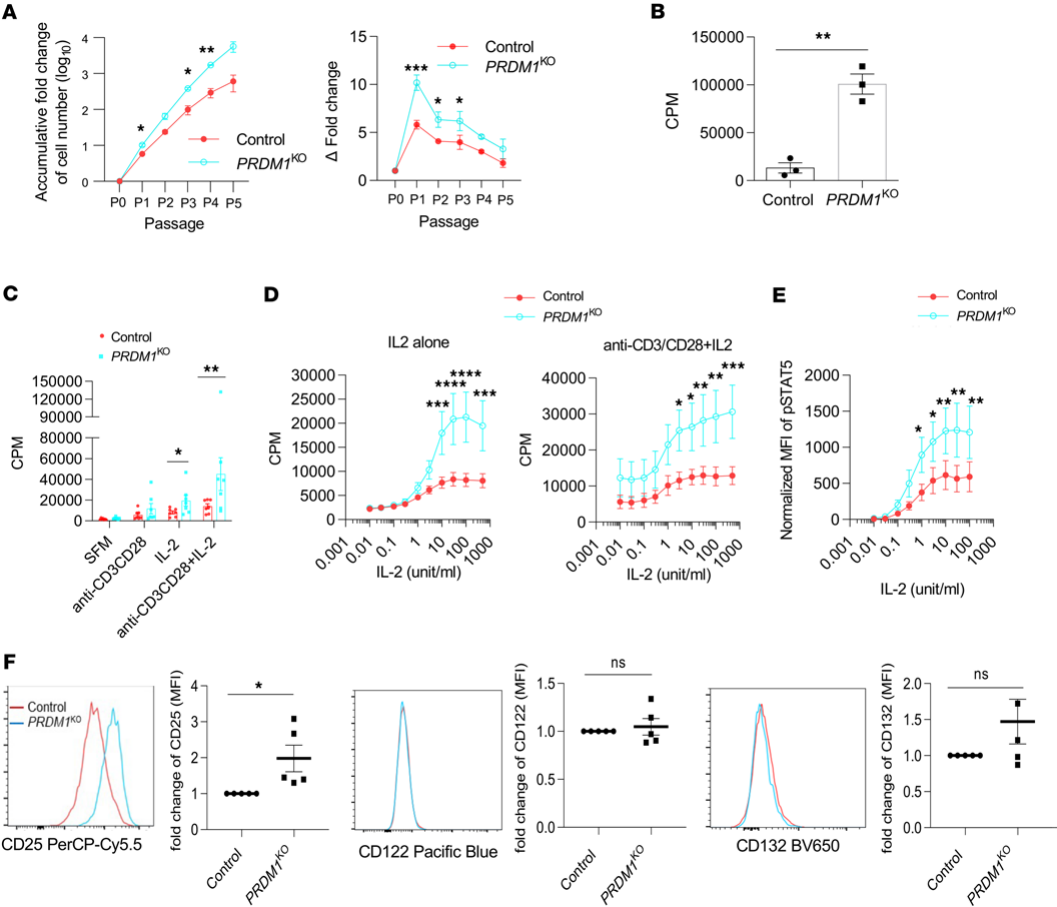
BLIMP-1 limits proliferation of human Tregs. (**A**) Control and *PRDM1*^KO^ Tregs (*n* = 3) were cultured for 3 days after electroporation, subcultured every 2 days at 2 × 10^5^ cells/mL with IL-2, and counted before each passage. Total (left) and per-passage (right) expansion are shown. (**B**) At 3 days after transfection, Tregs (5 × 10^4^/well; *n* = 3) were cultured for 4 additional days, where [^3^H]-thymidine was added during the last 4 hours of culture. (**C** and **D**) Control and *PRDM1*^KO^ Tregs (*n* = 7) were stimulated as indicated with 500 U/mL (**C**) or with various concentrations (**D**) of IL-2 for 3 days; cell proliferation was assessed by [^3^H]-thymidine incorporation. CPM, counts per million. (**E**) At 7 days after transfection, the indicated Tregs (*n* = 5) were rested for 4 hours and stimulated with the indicated concentrations of IL-2 for 15 minutes and pSTAT5 was enumerated. (**F**) IL-2R subunit expression was evaluated by flow cytometry on day 7; representative histograms and quantitative data (*n* = 5). Data are shown as the mean ± SEM and analyzed by 2-way ANOVA with multiple comparisons (**A** and **C**–**E**), or a paired 2-tail *t* test (**B**), or a 1-sample 2-sided *t* test (**F**). *P < 0.05, **P < 0.01, ***P < 0.001, ****P < 0.0001.

Based on GSEA results, we assessed the role of BLIMP-1 in human Treg growth by comparing responses by control and *PRDM1*^KO^ Tregs after CRISPR/Cas9 gene editing using control scrambled or *PRDM1* sgRNAs (Figure 1A). Treg expansion was greater at each time point by *PRDM1*^KO^ Tregs after serial passage with IL-2 (Figure 2A). Consistent with this finding, on day 7 posttransfection, *PRDM1*^KO^ Tregs showed greater proliferation as measured by [^3^H]-thymidine incorporation into DNA (Figure 2B).

To determine the influence of TCR and IL-2 stimulation on human Treg proliferation, control and *PRDM1*^KO^ Tregs on day 7 posttransfection were recultured with anti-CD3/CD28, IL-2, or their combination. Significant increases in proliferative responses were noted when *PRDM1*^KO^ Tregs were stimulated with IL-2 or IL-2 plus anti-CD3/CD28 (Figure 2C). The proliferation of *PRDM1*^KO^ Tregs to IL-2 or IL-2 plus anti-CD3/CD28 was enhanced to a similar magnitude in an IL-2 dose-dependent manner (Figure 2D), suggesting that BLIMP-1 normally contributes to restraining Treg proliferation to IL-2.

Given the increased proliferative response to IL-2 by *PRDM1*^KO^ Tregs, IL-2–dependent tyrosine phosphorylation of STAT5 (p-STAT5) was examined for control and *PRDM1*^KO^ Tregs. A much higher induction of p-STAT5 was noted for *PRDM1*^KO^ Tregs over an extended concentration of IL-2 (Figure 2E). This increase in p-STAT5 was associated with increased cell surface expression of CD25, but not CD122 and CD132 (Figure 2F). These results are in line with the higher sensitivity of cells, including Tregs, that is directly associated with increased cell surface amounts of CD25 (28, 29). Thus, BLIMP-1 normally downregulates CD25 in human Tregs to restrain their ability to proliferate to IL-2 and likely reflects that some IL-2–STAT5–dependent genes are negatively regulated by BLIMP-1 (Figure 1F).

### BLIMP-1 is independent of FOXP3 expression but required for Treg function.

**Figure 3:**
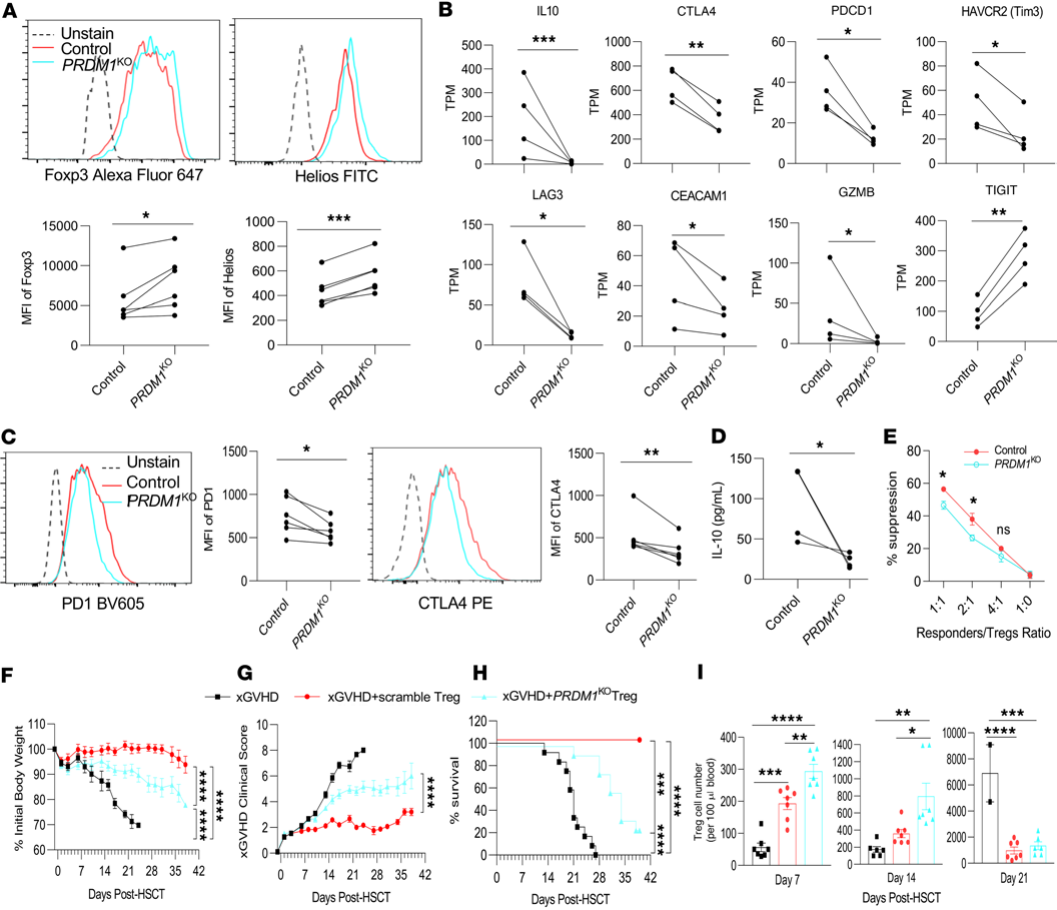
BLIMP-1 is required for optimal Treg function. (**A**–**C**) At 7 days after transfection with Cas9 RNP, the indicated Tregs were stimulated with anti-CD3/CD28 and IL-2 and assayed 16 hours later. (**A**) FOXP3 and HELIOS expression by flow cytometry (*n* = 6). (**B**) RNA expression by RNA-Seq of the indicated transcripts (*n* = 4). RNA data are expressed as transcripts/million (TPM). (**C**) PD1 and CTLA4 expression by flow cytometry (*n* = 6). (**D**) IL-10 secretion quantified by ELISA after PMA and ionomycin stimulation on day 7 posttransfection (*n* = 4). (**E**) In vitro suppression assay with control and *PRDM1*^KO^ Tregs (*n* = 3). (**F**–**I**) Effect of *PRDM1*^KO^ Tregs on the development of xGVHD. Irradiated NSG mice were adoptively transferred with PBMCs to induce xGVHD or in combination with control scramble or with *PRDM1*^KO^ Tregs, as indicated. xGVHD was assessed by weight loss (**F**), clinical scores (**G**), and overall survival (**H**) (*n* = 12 from 2 independent experiments). HSCT, hematopoietic stem cell transplantation. (**I**) Treg cell numbers in the blood were assessed at indicated time points after transplantation. Data are shown as the mean ± SEM and analyzed by a paired 2-tail *t* test (**A**–**D**), 2-way ANOVA with multiple comparisons (**E**), AUC with 1-way ANOVA with multiple comparisons (**F** and **G**), a log-rank (Mantel-Cox) test (**H**), or 1-way ANOVA with multiple comparisons (**I**). *P < 0.05, **P < 0.01, ***P < 0.001, ****P < 0.0001.

Recent work indicates that BLIMP-1 indirectly supports the stability of Tregs in an inflammatory environment by reducing DNA methyltransferase 3A (DNMT3A) methylation of *Foxp3* conserved non-coding sequence 2 (30). Indeed, human *PRDM1*^KO^ Tregs expressed an increased amount of DNMT3A RNA (Supplemental Figure 3A). Therefore, we examined the expression of FOXP3 and HELIOS (Ikaros family zinc finger protein 2), as their reduction is an indication of impaired stability. However, the absence of BLIMP-1 did not lower, but rather slightly increased *FOXP3* and *HELIOS* mRNA (Supplemental Figure 3B) and protein (Figure 3A). Consistent with this finding, control Tregs showed very few cells (<6%) that expressed IFN-γ, IL-4, IL-2, and IL-17A, and after ablation of *PRDM1*, the expression of these cytokines was reduced or unaffected (Supplemental Figure 3C), consistent with highly stable Tregs. Thus, under these culture conditions without the addition of inflammatory cytokines, these data indicate that BLIMP-1 does not contribute to FOXP3 and HELIOS expression and suggest that human Treg stability is independent of BLIMP-1.

Tregs exert their suppressive function through multiple mechanisms, including secretion of inhibitory cytokines, contact-dependent modulation through checkpoint proteins, and killing of target cells via granzyme. During in vitro expansion, control and *PRDM1*^KO^ Tregs acquired a phenotype of effector/activated Tregs (eTregs) based on uniform lack of expression of CD45RA and induction of CD69 and equivalent expression of CD39, CD73, and BCL-2 (Supplemental Figure 4A). Consistent with this, the expression of several mRNAs associated with eTregs, i.e., *IL10*, *CTLA4*, *PDCD1*, *TIM3*, *LAG3*, *CEACAM1*, and *GZMB*, decreased, while *TIGIT* increased, in *PRDM1*^KO^ Tregs (Figure 3B). Correspondingly, cell-associated PD-1 and CTLA4 (Figure 3C) and IL-10 secretion (Figure 3D) were reduced in BLIMP-1–deficient Tregs. *PRDM1*^KO^ Tregs also showed a modest, but significant, reduction in suppressive activity in vitro (Figure 3E and Supplemental Figure 4B). Thus, BLIMP-1 controls the development of eTregs and their function by multiple mechanisms.

To assess the role of BLIMP-1 function in vivo, the capacity of control scrambled versus *PRDM1*^KO^ Tregs to suppress xGVHD induced by human PBMCs was compared after adoptive transfer into humanized NOD/SCID-γ chain–deficient (NSG) mice. The *PRDM1*^KO^ Tregs exhibited a reduced ability to limit xGVHD when compared with control scrambled Tregs as assessed by increased loss in body weight (Figure 3F), increased clinical score (Figure 3G), and reduced overall survival (Figure 3H). These effects were noted even though the engraftment of the *PRDM1*^KO^ Tregs was significantly greater than the control cells 7 and 14 days after transfer (1.5- and 2.2-fold, respectively) (Figure 3I). In addition, human *PRDM1*^KO^ Tregs in the blood of NSG recipients showed increased expression of Ki67 on days 7 and 14, while BCL-2 was unaffected (Supplemental Figure 5). These findings are consistent with the in vitro studies, where *PRDM1*^KO^ Tregs showed increased proliferative capacity (Figure 2, A–E). Overall, these data in NSG mice demonstrate that BLIMP-1 is required for optimal human Treg suppressive activity while simultaneously restraining Treg expansion in vivo.

### BLIMP-1–dependent CEACAM1 is more highly induced in human Tregs.

**Figure 4:**
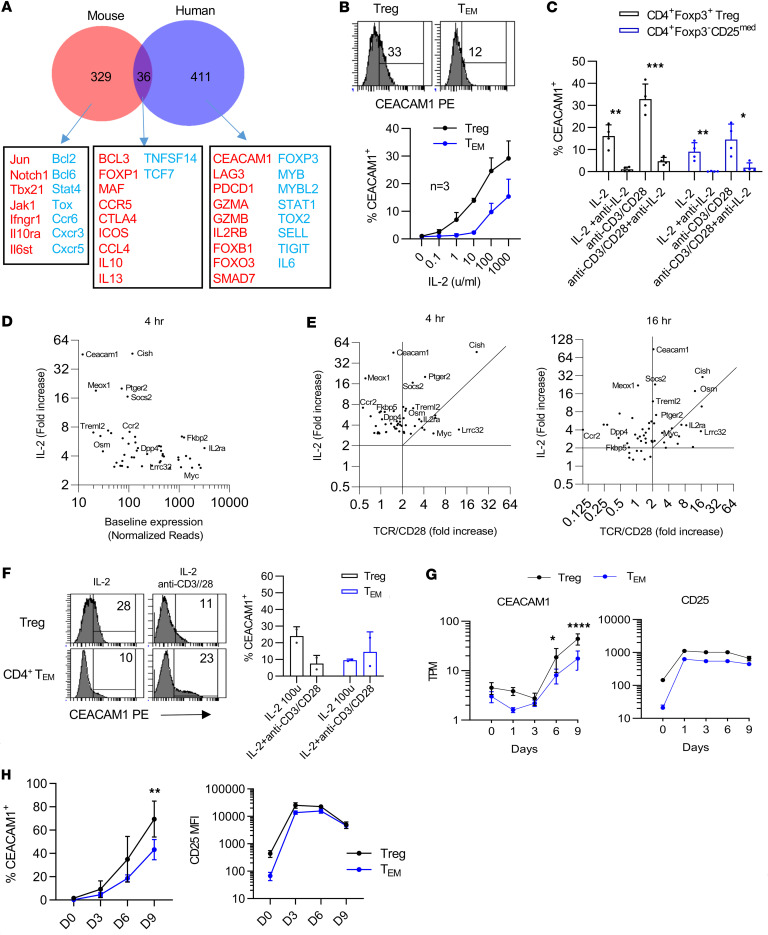
CEACAM1 expression is IL-2 dependent and more robust in human Tregs than Teff cells. (**A**) BLIMP-1–dependent DEGs identified in human Tregs (Figure 1D) were compared with Blimp-1–regulated DEGs in mouse Tregs. Venn diagram shows overlapping genes (≥1.5-fold change, *P* < 0.05); BLIMP-1–activated genes in red, repressed in blue. (**B**) Human CD4^+^ T cells were cultured for 2 days with IL-2; CEACAM1 expression was measured in Tregs (CD4^+^Foxp3^+^) and T_EM_ cells (CD4^+^Foxp3^–^CD25^med^) by flow cytometry; representative histograms (100 U/mL) and quantification (*n* = 3, mean ± SEM) are shown. (**C**) CD4^+^ T cells were cultured with IL-2 or anti-CD3/CD28 ± anti-IL-2; CEACAM1 expression was examined on gated Tregs and Teff cells (CD4^+^Foxp3^–^) (*n* = 4, mean ± SEM; paired 2-sided *t* test). (**D** and **E**) FACS-sorted CD4^+^CD25^hi^CD127^lo^ Tregs were cultured in media (*n* = 6), IL-2 (100 U/mL) (*n* = 4), or anti-CD3/CD28 with anti–IL-2 (TCR/CD28) (*n* = 6) for 4 or 16 hours. (**D**) RNA-Seq identified IL-2–induced DEGs (≥3-fold, FDR < 0.01) at 4 hours compared with basal expression in media. (**E**) Comparison of IL-2–dependent DEGs in **D** in relationship to the fold-change observed after culture with IL-2 versus TCR/CD28 at 4 and 16 hours. (**F**) FACS-sorted Tregs and CD4^+^CD45RA^–^CD25^med^CD127^hi^ T_EM_ cells were cultured for 5–6 days with IL-2 ± anti-CD3/CD28. CEACAM1 expression was determined by flow cytometry; representative histograms and quantification (*n* = 2, mean ± SEM). (**G** and **H**) Purified Tregs and T_EM_ cells were activated by anti-CD3/CD28 and IL-2 on day 0 and subcultured with only IL-2 on days 3 and 6. CEACAM1 and CD25 RNA (**G**) or surface protein (**H**) were determined by RNA-Seq or flow cytometry (*n* = 4, mean ± SEM; 2-way ANOVA). *P < 0.05, **P < 0.01, ***P < 0.001, ****P < 0.0001.

RNA-Seq revealed that several immune checkpoints require BLIMP-1 for optimal expression (Figure 1D and Figure 3B). One of these was *CEACAM1*, whose expression was 1.8-fold higher in control-treated Tregs (Figure 3B). Moreover, comparison of gene expression of mouse and human Tregs revealed that this appeared to be a species-specific effect, as we found that BLIMP-1 deficiency affected distinct sets of genes in humans and mice, with CEACAM1 emerging as the emblematic example (Figure 4A) (31). As the role of CEACAM1 has not yet been studied in Tregs, we further examined its function and relationship to BLIMP-1 and IL-2R signaling. First, we determined the requirements for CEACAM1 expression in human CD4^+^Foxp3^+^ Tregs and CD4^+^ T effector memory (T_EM_) cells, which were defined as CD4^+^Foxp3^–^CD25^med^ T cells (29). IL-2–induced CEACAM1 expression was ~2.5-fold greater on Tregs than T_EM_ cells in a dose-dependent manner (Figure 4B). This experiment also revealed that CEACAM1 was readily detected on only Tregs at low concentrations of IL-2 (1–10 U/mL) (Figure 4B). In addition, to achieve an equivalent percentage of CEACAM1^+^ T_EM_ cells, an approximately 50-fold higher amount of IL-2 was required (Figure 4B). When these CD4^+^ T cells were cultured solely with anti-CD3/CD28, more than 85% of CEACAM1 expression on Tregs and Teff cells was inhibited by anti–IL-2, indicating that endogenously produced IL-2 from conventional CD4^+^ T cells was controlling the expression of CEACAM1 in both cell types (Figure 4C). This finding is consistent with CEACAM1 being highly dependent on IL-2R, but not TCR, signaling.

To further examine the requirements for CEACAM1 expression, we mined bulk RNA-Seq data sourced from human Tregs (Supplemental Figure 6, A–C and G) cultured with IL-2. First, we identified 50 genes that were highly responsive to IL-2 (≥3-fold at 4 hours), including several well-characterized IL-2–dependent genes (*IL2RA*, *CISH*, *SOCS2*, *MYC*). Crucially, *CEACAM1* was the most IL-2–responsive gene (Figure 4D). It showed the lowest basal expression and the highest fold upregulation by IL-2 (Figure 4D). Given that many IL-2–responsive genes are also subject to TCR signaling, we next sought to determine if that is the case for CEACAM1. Strikingly, we found that when the induction of these 50 genes was examined in response to IL-2 versus TCR/CD28 stimulation at 4 and 16 hours, CEACAM1 showed high induction (>32-fold) with IL-2 and minimal activation by TCR/CD28 (~2-fold) (Figure 4E). Thus, CEACAM1 is among a small subset of genes that are largely dependent on IL-2 for their expression on Tregs.

Next, we compared CEACAM1 induction in Tregs with T_EM_ cells as both are antigen experienced and express the high-affinity IL-2R (29), albeit at a lower amount on T_EM_ cells. Tregs again showed a greater expression of CEACAM1 than Teff cells when cultured for 5–6 days with IL-2 (Figure 4F). In each of 2 experiments, IL-2–induced CEACAM1 was diminished when Tregs were concurrently stimulated with anti-CD3/CD28 plus IL-2. This effect was variable for T_EM_ cells. We do not know the precise mechanism for this effect. One possibility is that it might reflect consumption of IL-2 after activating Tregs in cultures following anti-CD3/CD28 stimulation, which induced high amounts of the IL-2R that lower the availability of IL-2 to promote CEACAM1 expression.

To quantify CEACAM1 expression over time more carefully, purified Tregs (Supplemental Figure 6, A–C and G) or T_EM_ (Supplemental Figure 6, D–G) cells were activated with anti-CD3/CD28 plus IL-2 and were subcultured with only IL-2 on days 3 and 6. A low amount of *CEACAM1* mRNA was noted on days 1 and 3 (Figure 4G), and minimal CEACAM1 surface protein was detected by flow cytometry (Figure 4H) on both cell types on day 3. However, after subculture with only IL-2, expression of CEACAM1 was readily detected, and these amounts were approximately 1.5-fold greater for Tregs (Figure 4H and Supplemental Figure 7). This finding and the mRNA results (Figure 4G) are consistent with IL-2 rather than TCR/CD28 signaling inducing CEACAM1 expression. In contrast with CEACAM1, CD25 mRNA and surface protein were rapidly upregulated over this time course (Figure 4, G and H, and Supplemental Figure 7). Collectively, these data demonstrate that CEACAM1 is an IL-2–dependent activation molecule that is more prominently induced in Tregs when compared with activated T_EM_ cells.

### CEACAM1 as an IL-2–dependent biomarker for low-dose IL-2 therapy.

**Figure 5:**
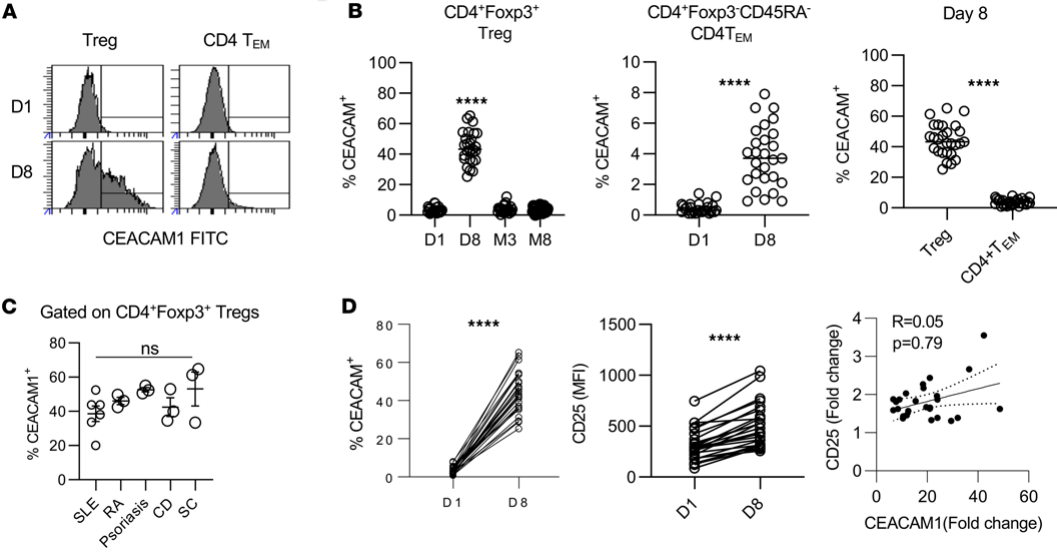
Low-dose IL-2 upregulates CEACAM1 in Tregs from autoimmune patients. A total of 27 patients with 8 autoimmune diseases (SLE *n* = 6; RA, psoriasis, CD, SC, ankylosing spondylitis, Sjögren’s syndrome, and SSc *n* = 3 each) received human IL-2 (1 million IU/d) from day 1–5 (induction), then biweekly from day 15–180 (maintenance). Samples were collected prior to treatment (D1), 3 days after induction period (D8), at month 3 (M3) prior to the maintenance injection, and at month 8 (M8), 2 months after the completion of therapy. (**A**) Representative histograms of CEACAM1 expression in CD4^+^ Tregs or T_EM_ cells at the indicated time points. (**B**) Quantification of CEACAM1 expression in Tregs and CD4^+^CD45RA^–^ T_EM_ cells from all patients. Data show the mean and were analyzed by unpaired 2-sided *t* test. (**C**) CEACAM1 expression in Tregs at day 8 by disease group (mean ± SEM, 1-way ANOVA). (**D**) The relationship between CEACAM1 and CD25 expression. Fold change for the percentage of CEACAM1^+^ Tregs (left) and MFI of CD25 expression (middle) after low-dose IL-2, with correlation across patients analyzed by Spearman’s rank method (right). ****P < 0.0001.

To assess the relevance of IL-2R regulation of CEACAM1 in vivo, we examined the expression of CEACAM1 upon low-dose IL-2 therapy in 27 patients with 8 autoimmune diseases, i.e., systemic lupus erythematosus (SLE), rheumatoid arthritis (RA), psoriasis, Crohn’s disease (CD), sclerosing cholangitis (SC), ankylosing spondylitis, Sjögren’s syndrome, and systemic sclerosis (SSc) (32). These patients initially received 1 × 10^6^ international units (IU) of human IL-2 s.c. each day for 5 days and then every 15 days for 6 months. When compared with baseline (day 1), CEACAM1 was highly induced in Tregs when examined 3 days after the 5-day induction course of low-dose IL-2 (day 8) (Figure 5, A and B). This expression was variable (range 28.8%–64.7%) and transient, as CEACAM1 returned to baseline levels when measured at 3 months, 2 weeks after the previous maintenance injection, or 2 months after low-dose IL-2 therapy ceased (month 8) (Figure 5B). CEACAM1 was induced on average to an 11.7-fold higher percentage of Tregs (range 25.3%–65.3%) than on CD4^+^CD45RO^+^ T_EM_ cells (range 0.9%–7.0%) on day 8 (Figure 5B), consistent with selectivity of low-dose IL-2 toward Tregs. In addition, when compared with baseline levels, CEACAM1 increased 13.8-fold on Tregs and 8.7-fold on CD4^+^CD45RO^+^ T_EM_ cells (Figure 5B). The variable induction of CEACAM1 in Tregs by low-dose IL-2 was not resolved when considering each autoimmune disease separately (Figure 5C). This may reflect the low number of patients in each disease or some other yet-to-be-defined factors that variably contributed to IL-2–dependent CEACAM1 expression. In striking contrast with human Tregs, CEACAM1 expression was not detected on mouse Tregs prior to or after using the IL-2 agonist, IL-2/CD25 fusion protein (Supplemental Figure 8). Thus, CEACAM1 is likely uniquely regulated by IL-2 in human Tregs.

The percentage of CEACAM1 and the MFI of CD25 (Figure 5D), a TCR- and IL-2–induced protein, was determined for Tregs relative to baseline for each patient. Surprisingly, the upregulation of CEACAM1 and CD25 on Tregs showed no correlation (Figure 5D). This result indicates that distinct requirements contribute to the IL-2R–dependent expression of CD25 versus CEACAM1, most likely because the former also depends on TCR signaling. In contrast, consistent with the selectivity of low-dose IL-2 for Tregs and analogous to CEACAM1 (Figure 5B), CD25 amounts were minimally affected by low-dose IL-2 for CD4^+^ T_EM_ cells (Supplemental Figure 9). Collectively, these data indicate that CEACAM1 is a biomarker that is highly specific to IL-2 with preferential activity in Tregs.

### Optimal CEACAM1 expression in human Tregs depends upon chromatin remodeling driven by IL-2 and BLIMP-1.

**Figure 6:**
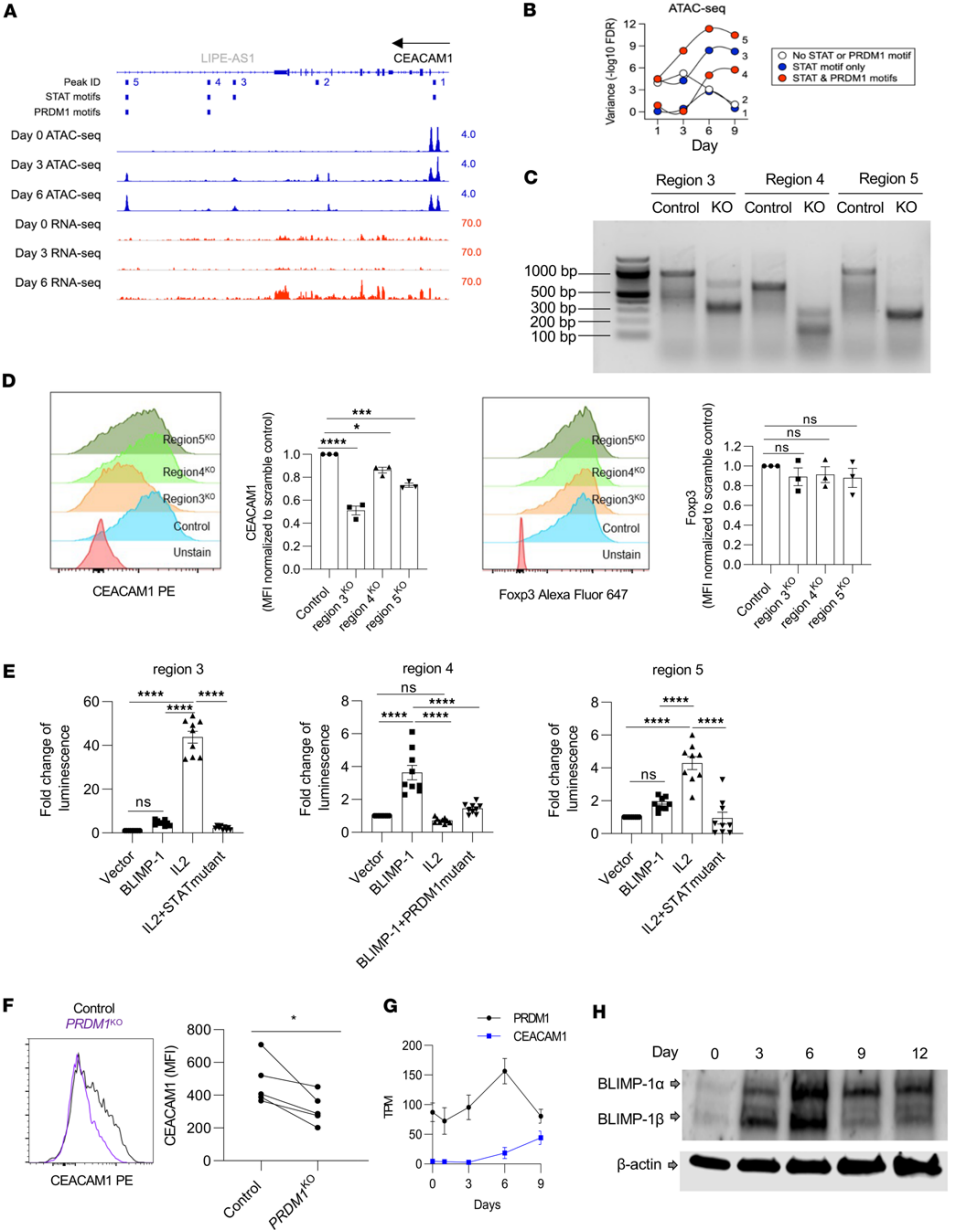
IL-2R–dependent CEACAM1 expression depends on chromatin opening and BLIMP-1. (**A**) Purified human Tregs were stimulated with anti-CD3/CD28 and IL-2, then subcultured with IL-2 alone on days 3 and 6. ATAC-Seq and RNA-Seq were performed at the indicated times, and Genome browser tracks were plotted for the CEACAM1 locus. Peaks with STAT and PRDM1 motifs were identified using Meme suite 5.4.1. Peak IDs refer to regions with substantial changes in sequence reads, showing peaks with *STAT* and *PRDM1* motifs. (**B**) Variance in accessibility across peaks over time is shown. (**C** and **D**) Purified Tregs stimulated for 3 days with anti-CD3/CD28 plus IL-2 were transfected with scramble or Cas9 RNPs targeting accessible regions (regions 3–5) and cultured for 7 more days with IL-2. (**C**) T7EI assay confirmed editing at the targeted region. Expected PCR product sizes are 928 bp (region 3), 663 bp (region 4), and 1,036 bp (region 5). (**D**) CEACAM1 and Foxp3 expression was analyzed by flow cytometry; representative and quantitative data (*n* = 3). (**E**) HEK-Blue IL-2 cells were cotransfected with luciferase reporters driven by the indicated wild-type or mutated CEACAM1 regions and either control or PRDM1 vectors. Cells were treated ± IL-2 (500 U/mL) for 24 hours, and luciferase activity was measured and normalized to baseline. (**F**) CEACAM1 expression was assessed by flow cytometry; representative histograms and quantitative data (*n* = 5). (**G** and **H**) Purified Tregs were initially stimulated with anti-CD3/CD28 and IL-2 and subcultured with IL-2 on days 3 and 6. (**G**) Time course of *PRDM1* and *CEACAM1* mRNA expression by RNA-Seq. (**H**) Expression of BLIMP-1 during expansion was examined by Western blotting analysis. Data were analyzed by 1-way ANOVA (**D** and **E**) or paired 2-sided *t* test (**F**). *P < 0.05, ***P < 0.001, ****P < 0.0001.

To better understand the mechanism by which CEACAM1 expression is regulated, the effect of TCR/CD28 and IL-2 on *CEACAM1* chromatin accessibility was assessed by ATAC-Seq. Five regions of chromatin accessibility were detected within the greater *CEACAM1* locus (Figure 6A). Region 1 comprises 2 adjacent peaks and is just upstream the *CEACAM1* start site, which likely defines the promoter. This was the only region found to be accessible at baseline in unstimulated Tregs. Region 2 is within an intron while regions 3–5 are downstream of the last exon of *CEACAM1*. All 4 of these regions became accessible by 3 days after IL-2 exposure, and this state was maintained through day 6. Crucially, the opening of these 4 regions on day 3 preceded accumulation of high amounts of *CEACAM1* mRNA and protein (Figure 4, G and H), which became evident starting on day 6.

Importantly, ATAC-Seq peaks 3–5 had far less variance than peaks 1–2, suggesting they are the most robustly affected by IL-2. These were also the only peaks that harbored both *STAT* and *PRDM1* consensus DNA motifs (Figure 6B). To determine the relevance of these regions to CEACAM1 expression, CRISPR/Cas9 editing was performed to disrupt the approximately 400 bp sequences that comprised peaks 3–5. High editing efficiency was observed as the wild-type bands associated with peaks 3–5 were undetectable for each peak as assessed by the T7EI mismatch assay (Figure 6C). The expression of CEACAM1 and Foxp3 was assessed 7 days after CRISPR/Cas9 editing. A marked reduction of CEACAM1, but not Foxp3, was observed after the individual disruption of peaks 3–5. This effect was most striking for peaks 3 and 5, where CEACAM1 expression was reduced by 49% and 27%, respectively (Figure 6D). Thus, these data are consistent with the increased accessibility associated with the differentially assessable regions regulating the expression of *CEACAM1*.

To test whether STAT5 and BLIMP-1 regulate the expression of *CEACAM1*, DNA sequences of regions 3, 4, and 5 were each linked to a luciferase reporter construct, and these were transfected into HEK-Blue IL-2 cells. These are HEK293 cells that were previously stably transfected with human CD25, CD122, CD132, JAK3, and STAT5 and responded to IL-2 as measured by secretion of embryonic alkaline phosphatase linked to a STAT5-inducible reporter gene. When these cells were transiently transfected with the luciferase reporters and stimulated with IL-2 or cotransfected with a BLIMP-1 expression vector, regions 3 and 5 were responsive to IL-2 due to endogenous STAT5 whereas region 4 was responsive to BLIMP-1 (Figure 6E). The amount of luciferase induction was directly proportional to the reduction in cell surface CEACAM1 expression when each region was ablated by CRISPR/Cas9 targeting (Figure 6D). These findings are consistent with IL-2 and BLIMP-1 directly contributing to positive *CEACAM1* gene expression.

*PRDM1* is an IL-2– and STAT5-dependent gene whose expression is associated with eTregs (17, 18, 33, 34), including terminally differentiated KLRG1^+^ Tregs that have extensively expanded in response to IL-2. Since the high expression of *CEACAM1* mRNA and protein was detected after considerable IL-2–dependent expansion (Figure 4, G and H) and BLIMP-1 promoted CEACAM1 through *PRDM1* motifs in region 4 (Figure 6E), we further assessed whether CEACAM1 depends on BLIMP-1. Flow cytometry revealed that surface expression of CEACAM1 was reduced on average by 1.5-fold in the absence of BLIMP-1 (Figure 6F). Moreover, optimal *PRDM1* mRNA and protein expression (day 6) preceded that of *CEACAM1* (day 9) (Figure 4H and Figure 6, G and H). Overall, we observed that CEACAM1 mRNA (Figure 3B) or surface protein (Figure 6F) was reduced in Tregs from 9 humans after *PRDM1* KO. Thus, BLIMP-1 is required for optimal expression of CEACAM1 and, together with STAT5, contributes to chromatin accessibility and transcription of that locus.

### CEACAM1 is a TCR checkpoint in Tregs.

**Figure 7:**
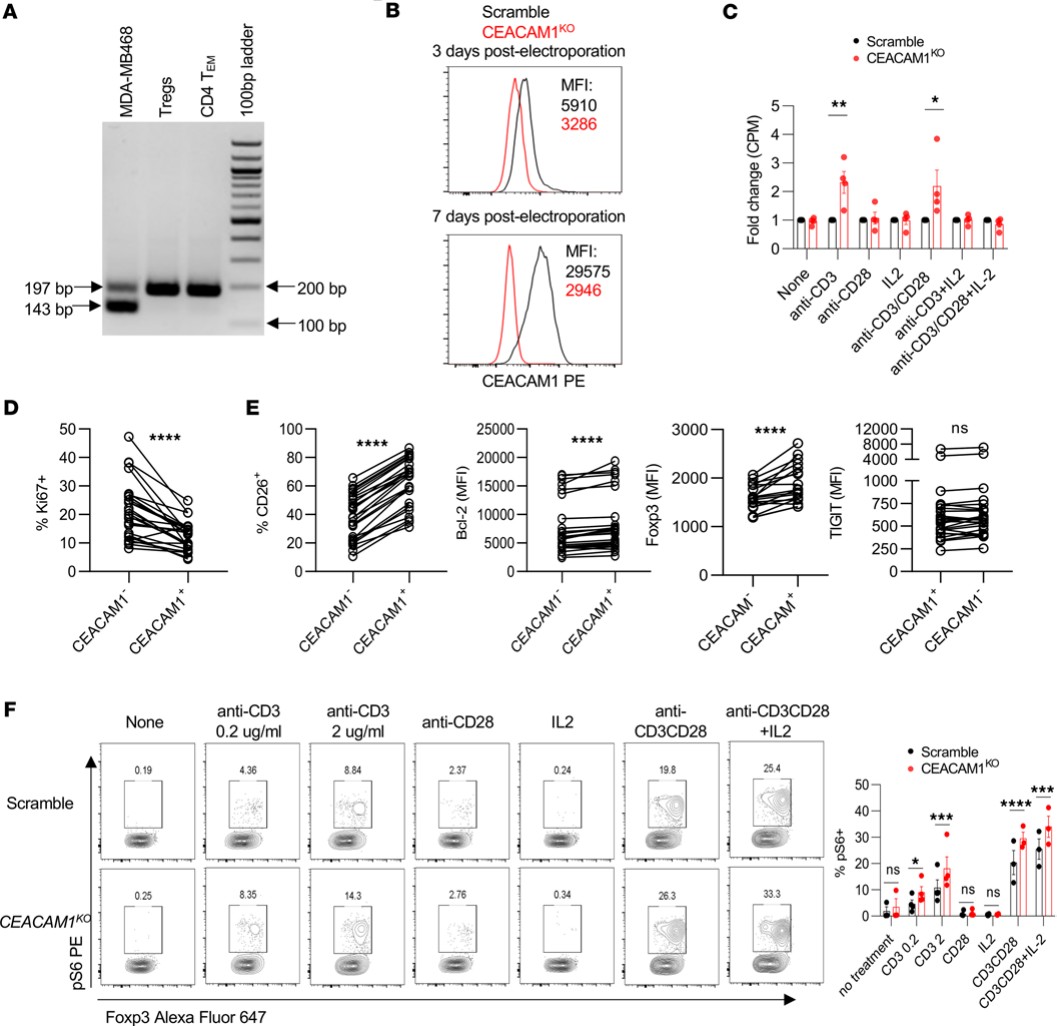
CEACAM1 is a TCR checkpoint in Tregs. (**A**) RT-PCR showing CEACAM1 long (197 bp) and short (143 bp) isoforms. (**B** and **C**) Purified Tregs were stimulated with anti-CD3/CD28 and IL-2 for 3 days, electroporated with scramble or CEACAM1-targeting Cas9 RNPs, and then subcultured with IL-2 for 7 days. (**B**) Representative histograms of CEACAM1 expression with MFI values. (**C**) Proliferation assessed by ^3^H-thymidine incorporation after 3-day restimulation on scramble and *CEACAM1*^KO^ Tregs. Data were normalized to the scramble control and shown as quantitative data of fold change (*n* = 4 biological replicates; mean ± SEM; 1-sample 2-sided *t* test). (**D** and **E**) CEACAM1^+^ and CEACAM1^-^ Tregs from low-dose IL-2–treated patients (see legend to Figure 5) on day 8 were analyzed for proliferation (**D**) and immune markers (**E**) (paired 2-sided *t* test). (**F**) After 7-day IL-2 expansion, scramble and *CEACAM1*^KO^ Tregs were rested overnight and treated for 6 hours as indicated. Representative plots and quantitative data of pS6 activation (*n* = 3; mean ± SEM; 2-way ANOVA). *P < 0.05; **P < 0.01; ***P < 0.001; ****P < 0.0001.

The long isoform of CEACAM1, which limits TCR signaling in activated conventional T cells through ITIMs associated with its cytoplasmic tail (8, 23, 35), was readily and exclusively detected in Tregs and T_EM_ cells after anti-CD3/CD28 plus IL-2 activated cells were subcultured with only IL-2 (Figure 7A). To directly assess the role of CEACAM1 in Tregs, *CEACAM1* was ablated using the same approach for *PRDM1* (Figure 1A) but using guides for *CEACAM1*. Purified Tregs were activated with anti-CD3/CD28 plus IL-2, and 3 days later, guide RNAs to exons 2 and 4 of *CEACAM1* (Supplemental Figure 10A) were used to disable the *CEACAM1* locus by CRISPR/Cas9 editing. High knockout efficiency was achieved as measured by T7EI mismatch assay (>98% on exon 2 and >69% on exon 4, respectively; Supplemental Figure 10B) and Western blotting (Supplemental Figure 10C). Moreover, flow cytometry showed that CEACAM1 expression was reduced on day 3 and essentially undetectable on day 7 after CRISPR editing (Figure 7B).

We tested the role of CEACAM1 on Treg proliferation and function. With respect to proliferation, as expected, the greatest proliferation occurred when Tregs were stimulated with anti-CD3/CD28 and IL-2, and these responses were typically similar for wild-type and *CEACAM1*^KO^ Tregs (Supplemental Figure 11). However, in each of the 4 independent experiments, when IL-2–expanded Tregs were restimulated with only anti-CD3 or anti-CD3/CD28, *CEACAM1*^KO^ Tregs exhibited increased proliferation (Supplemental Figure 11). On average these proliferative responses increased 2.2- to 2.3-fold (Figure 7C).

These in vitro studies showed that CEACAM1 limits the proliferative response of Tregs to TCR/CD28 stimulation (Figure 7C). Accordingly, we assessed the proliferative state of CEACAM1-bearing Tregs in patients undergoing low-dose IL-2 therapy. On day 8, 3 days after the 5-day administration of low-dose IL-2, Tregs that had upregulated CEACAM1 exhibited a lower proliferative response than those that were CEACAM1^–^ Tregs based on Ki67 expression (Figure 7D and Supplemental Figure 12). Thus, CEACAM1 expression is associated with lower Treg proliferation in vivo. CEACAM1^+^ Tregs expressed greater amounts of CD26, BCL-2, and Foxp3, but not TIGIT (Figure 7E and Supplemental Figure 12), where the former 3 are known to be positively regulated by IL-2 (29). Thus, the findings from *CEACAM1*^KO^ Tregs and Tregs from patients undergoing low-dose IL-2 therapy support the notion that CEACAM1 functions as a Treg checkpoint.

The mechanism by which CEACAM1 affects T cell proliferation was assessed by examining TCR and IL-2R signaling in wild-type and *CEACAM1*^KO^ Tregs. Anti-CD3 activation of *CEACAM1*^KO^ Tregs led to a greater activation of pS6, downstream of AKT and mTOR complex 1, when compared with wild-type Tregs (Figure 7F). These data indicate CEACAM1 functions to limit TCR signaling. In contrast, CEACAM1 showed only a slightly lower response to IL-2 as assessed by IL-2–dependent activation of p-STAT5 (Supplemental Figure 13A), as on average the EC_50_ of WT versus CEACAM1^KO^ Tregs increased from 0.56 to 1.1 U/mL, respectively. *CEACAM1*^KO^ Tregs showed a slight decrease in expression of CD25, but not CD122 and CD132 (Supplemental Figure 13B), which likely accounts for this result. However, this small difference in IL-2R sensitivity did not affect Treg proliferation to IL-2 (Figure 7C) because the Tregs were cultured with a high amount (500 U/mL) of IL-2. Overall, these findings indicate CEACAM1 limits Treg proliferation to TCR signaling.

### CEACAM1 limits the function of human Tregs in vivo.

**Figure 8:**
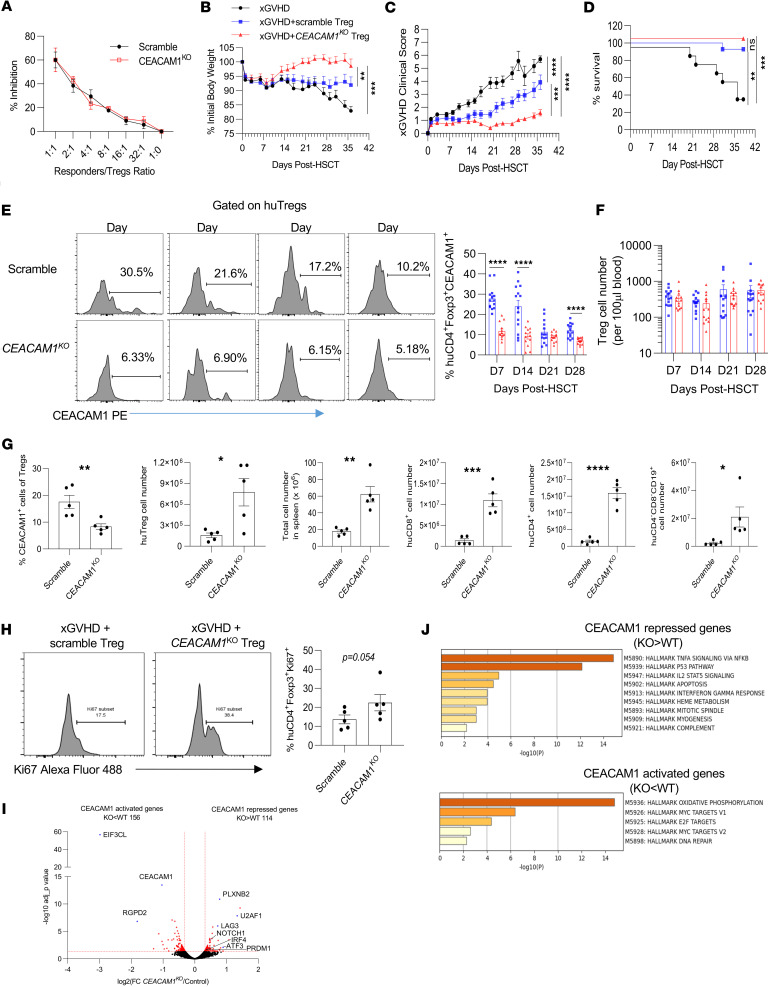
CEACAM1 restrains the suppressive function of human Tregs in vivo. (**A**) The in vitro suppressive activity of scramble and *CEACAM1*^KO^ Tregs on day 5 after electroporation (*n* = 3; mean ± SEM). (**B**–**D**) Irradiated NSG mice were adoptively transferred with PBMCs or in combination with scramble or *CEACAM1*^KO^ Tregs (*n* = 10–14). xGVHD was assessed by body weight (**B**), clinical scores (**C**), and survival (**D**). (**E** and **F**) Blood was collected on day 7, 14, 21, and 28 after transplantation. CEACAM1 expression (**E**) and human Treg number (**F**) were measured by flow cytometry (*n* = 14). (**G** and **H**) On day 36, spleens were analyzed for CEACAM1 expression by Tregs and cell numbers of the indicated cell populations (**G**) and Ki67 expression by human Tregs (**H**) (*n* = 5). (**I** and **J**) RNA-Seq of donor control and *CEACAM1*^KO^ human Tregs before transplantation. (**I**) Volcano plot illustrating CEACAM1-activated and -repressed genes. DEGs with an expression difference of ≥1.25-fold and FDR value of <0.05 are colored in red. (**J**) Hallmark pathway analysis of DEGs. Data (**B**–**H**) are from 2 independent experiments and are analyzed using AUC with 1-way ANOVA (**B** and **C**), log-rank (Mantel-Cox) test (**D**), 2-way ANOVA with multiple comparisons (**E** and **F**), and unpaired 2-sided *t* test (**G** and **H**); *P < 0.05, **P < 0.01, ***P < 0.001, ****P < 0.0001.

With respect to Treg function, depletion of CEACAM1 on Tregs had little effect on suppressive activity in vitro (Figure 8A and Supplemental Figure 14). However, the standard in vitro suppression assay does not always reflect the function of Tregs in vivo (36), so we next assessed control scrambled versus *CEACAM1*^KO^ Tregs to suppress xGVHD induced by human PBMCs after adoptive transfer into humanized NSG mice. While donor Tregs expressed high amounts of Foxp3, cell surface CEACAM1 was much lower on *CEACAM1*^KO^ Tregs (average 13.3%) than control scrambled Tregs (average 83.4%) (Supplemental Figure 15A). This reduction reflected a 5.8-fold decrease in CEACAM1 based on the MFI of the total population of donor Tregs.

*CEACAM1*^KO^ Tregs exhibited enhanced capacity to limit xGVHD when compared with control scrambled Tregs, with better control of body weight (Figure 8B) and lower clinical GVHD score (Figure 8C), resulting in 100% survival at 6 weeks (Figure 8D). Since CEACAM1 limits Treg proliferation but did not lower Treg suppressive activity in vitro, we assessed whether increased proliferation of *CEACAM1*^KO^ Tregs accounts for the enhanced suppression of xGVHD. Analysis of donor Tregs in the blood of NSG mice showed that recipients that received *CEACAM1*^KO^ Tregs had reduced CEACAM1 expression (Figure 8E) but similar Treg numbers compared with the control group (Figure 8F). However, at the conclusion of the experiment (day 36), spleens from mice receiving *CEACAM1*^KO^ Tregs had 6.6 times more Tregs than those from the control group (Figure 8G), with higher Ki67 expression (Figure 8H). These findings raise the possibility that the initial decreased expression of CEACAM1 by the knockout donor Tregs supported increased proliferation. Additionally, spleen cell cellularity was approximately 3.5-fold greater for NSG mice that received the *CEACAM1*^KO^ Tregs (Figure 8G). This increased engraftment corresponded to better overall health of these mice and included increases in CD4^+^ and CD8^+^ T cells, Tregs, and CD19^+^ B cells (Figure 8G). Overall, CEACAM1 deficiency increased Treg suppressive activity in vivo.

Bulk RNA-Seq was also performed on control scrambled and *CEACAM1*^KO^ donor Tregs prior to adoptive transfer. We found 270 genes were differentially expressed (>1.25-fold, FDR < 0.05) (Figure 8I). Similar to *PRDM1*^KO^ Tregs (Figure 1E), IFN signaling was enriched in *CEACAM1*^KO^ Tregs relative to controls (Figure 8J). A small but significant increase in *PRDM1* and *IRF4* was also detected for *CEACAM1*^KO^ Tregs (Figure 8I), but these Tregs did not display a gene signature indicative of enhanced Treg suppressive activity (Figure 8J and Supplemental Figure 15, B and C). In addition, genes associated with oxidative phosphorylation were reduced while genes related to p53 pathway were upregulated in *CEACAM1*^KO^ Tregs (Figure 8J and Supplemental Figure 15C). This phenotype may prime *CEACAM1*^KO^ Tregs to integrate in vivo signals, including those from the TCR and CD28, more effectively upon transfer to the humanized NSG mice. This benefit may be most critical early on as the high expression of CEACAM1 by the control scrambled Tregs (Supplemental Figure 15A) was downregulated to much lower levels when donor Tregs were examined in the blood 7-day posttransfer (Figure 8E). Collectively, our in vitro and in vivo findings suggest that CEACAM1 negatively regulates Treg suppressive function, at least in part by lowering TCR signaling in human Tregs.

## Discussion

BLIMP-1 controls a major transcriptional hub distinct from Foxp3 in human Tregs (26). We revealed that BLIMP-1 restrains Treg proliferation while supporting eTreg-associated gene expression. BLIMP-1 limits Treg proliferation by 2 complementary mechanisms: dampening IL-2 responsiveness by lowering CD25 expression and limiting TCR/CD28 signaling via CEACAM1 upregulation. High expressions of BLIMP-1 and CEACAM1 are downstream of and require sustained IL-2R signaling. BLIMP-1 also optimizes Treg function by upregulating 2 well-described Treg-dependent functional pathways, secretion of IL-10 and expression of CTLA4 in vitro.

Much of our knowledge concerning BLIMP-1 in T cells stems from mouse models. In conventional T cells, BLIMP-1 suppresses *IL-2* transcription to limit proliferation while promoting terminal differentiation of highly active CTLs (14, 37). BLIMP-1 also intrinsically controls mouse Treg homeostasis (16, 31), but the mechanism restraining their proliferation remains unclear. We show that BLIMP-1 also contributes to the development of activated human Tregs by upregulating functional molecules (IL-10, CTLA4, GZMB) and other T cell immune checkpoints. Unlike in conventional T cells, BLIMP-1 is dispensable for IL-2 production in Tregs, as this is repressed by Foxp3 (38). Instead, BLIMP-1 limits Treg proliferation through downregulation of CD25 and decreased IL-2R signaling. Consistent with findings in mice (39), we demonstrate that BLIMP-1 enhances IL-10 expression in human Tregs. Although BLIMP-1 is typically considered a transcriptional repressor (24, 40), it also sometimes acts as a gene activator, notably in cooperation with IRF4 to upregulate *IL-10* transcription in Tregs and other lymphoid cells (17).

Our findings related to BLIMP-1 in Treg proliferation in vitro suggest that BLIMP-1 is an important regulator of human Treg homeostasis and function. Indeed, *PRDM1*^KO^ Tregs exhibited increased expansion and reduced suppressive activity in a humanized xGVHD mouse model, indicating that BLIMP-1 restrains proliferation while promoting function. Several polymorphisms in *PRDM1* are risk alleles for ulcerative colitis, CD, SLE, and RA (41–44), although they have not been formally associated with impaired Tregs. Alternatively, the role of BLIMP-1 in human Tregs in vivo may be subtle, especially when considering its role in mouse Tregs. T cell–selective *PRDM1* deletion in mice caused early-onset colitis because of excessive CD4^+^ T cell proliferation (45), suggesting a major contribution for BLIMP-1 on Treg homeostasis and function. However, this phenotype was not observed when *PRDM1* was conditionally deleted in mouse Tregs (16, 31). In this setting, Treg homeostasis was affected with increased Treg numbers and a shift toward eTregs without impairing functional activity. In the absence of BLIMP-1 in only mouse Tregs, a lethal multiorgan inflammatory syndrome took over 1 year to develop (16), and IL-17 was activated in a subset of RORγt^+^ Tregs (46).

BLIMP-1 positively regulates several immune checkpoints in human Tregs, including CEACAM1, PD-1, and TIM3. Of these, little is known about the function of CEACAM1. In a mouse model of immune-mediated hepatitis and in patients with SLE, the short isoform of CEACAM1 was implicated in the conversion of CD4^+^ T cells into induced Tregs (47, 48). However, Tregs were not tested for expression of CEACAM1 protein; thus, it is unclear whether CEACAM1 regulates induced Treg differentiation at the level of activated conventional T cells or Tregs. Another study identified CEACAM1 expression on some highly activated Tregs in the tumor microenvironment of patients with renal cell carcinoma or ovarian cancer (49).

Based on these limited studies, we initially compared properties of CEACAM1 in Tregs versus T_EM_ cells, since the latter has been extensively studied (50). We found that CEACAM1 is more highly expressed in Tregs, with its expression primarily dependent on the IL-2R, rather than CD3/CD28, signaling. This finding indicates that CEACAM1 is more critical to regulate Tregs. Although short-term IL-2 stimulation revealed CEACAM1 as a direct IL-2–responsive gene, its mRNA and surface protein levels continued to substantially increase after subculture of the anti-CD3/CD28/IL-2–primed T cells with only IL-2. This response pattern aligns with the identification of CEACAM1^+^ Tregs in a tumor microenvironment dominated by activated Tregs (49). The mechanism for this delayed upregulation is likely related in part to chromatin remodeling, which increases opening of *CEACAM1* that precedes enhanced mRNA and protein levels. Indeed, 3 regions downstream (3′) of *CEACAM1* showed increased chromatin accessibility and contributed to CEACAM1 expression, as demonstrated by reduced expression following CRISPR/Cas9 disruption of these regions. Motif analysis identified *PRDM1* and *STAT* binding sites within these regions. *STAT* motifs in regions 3 and 5 and *PRDM1* motifs in region 4 enhanced luciferase reporter activity when linked to these putative enhancers. Moreover, *PRDM1* deficiency also diminished CEACAM1 expression. These findings are consistent with a direct role for BLIMP-1 and IL-2–activated STAT5 in promoting transcription in newly opened chromatin 3′ of *CEACAM1*.

CEACAM1 expression on T cells in autoimmune patients undergoing low-dose IL-2 therapy paralleled that found in vitro, with substantially higher expression on Tregs than T_EM_ cells after the 5-day induction treatment. This supports the selectivity of low-dose IL-2 on Tregs. Unlike CD25, which is induced by both TCR and IL-2R signaling (51), CEACAM1 is upregulated specifically by IL-2, making it a more precise marker of an IL-2 response. Indeed, linear regression analysis of CEACAM1 versus CD25 showed at best a weak relationship. Thus, the upregulation of CD25 during low-dose IL-2 may reflect both TCR and IL-2 while CEACAM1 more strictly reflects IL-2 activity.

The variability in CEACAM1 upregulation on Tregs during low-dose IL-2 therapy suggests patient-specific differences in CEACAM1 regulation. This heterogeneity does not appear linked to a particular autoimmune disease, although larger cohorts are needed to confirm this finding. Alternatively, it may reflect some intrinsic Treg property related to integration of IL-2R signaling. As such, following CEACAM1 during low-dose IL-2 may be a useful biomarker for predicting individual IL-2 responsiveness and therapeutic outcomes.

Analogous to Teff cells (8, 23, 52), our study revealed that CEACAM1 acts as a TCR checkpoint for Treg proliferation. Several findings support its role in limiting TCR signaling. First, activated Tregs exclusively expressed the long isoform of CEACAM1 that recruits SHP-1 to 2 ITIMs known to inhibit TCR signaling (23). Second, cultured *CEACAM1*^KO^ Tregs showed a higher proliferative response to TCR/CD28 restimulation. Third, *CEACAM1*^KO^ Tregs showed higher downstream TCR signaling, i.e., activation of pS6, while IL-2R signaling was minimally affected. Fourth, in autoimmune patients initially treated with low-dose IL-2, CEACAM1^+^ Tregs showed a lower proliferative response based on Ki67 expression, possibly reflecting interplay between TCR and IL-2 signaling. Last, *CEACAM1*^KO^ Tregs were more effective than scrambled Tregs in limiting xGVHD in humanized NSG mice. Aside from modestly increased *PRDM1* and *IRF4* expressions, which support eTregs, RNA-Seq of the input cells indicated that *CEACAM1*^KO^ Tregs were not programmed to be more suppressive. This result and the rapid CEACAM1 downregulation in control Tregs suggest that *CEACAM1*^KO^ Tregs may have more favorably integrated TCR signaling after transfer, enhancing their engraftment to more effectively control xGVHD. In contrast, *PRDM1*^KO^ Tregs were less suppressive in xGVHD, consistent with BLIMP-1’s role as a transcriptional hub that upregulates key suppressive gene expression, including CTLA4 and IL-10. CEACAM1, in contrast, is only one component of this hub that did not affect these mediators when deleted.

Overall, our findings reveal a sequential and negative feedback regulatory loop where IL-2–induced BLIMP-1 and CEACAM1 limit Treg responsiveness to IL-2R and TCR/CD28 signaling, likely impacting their homeostasis. Initially, TCR/CD28 activation of Tregs is facilitated by suppression of the TCR checkpoint CEACAM1. Persistent IL-2R signaling induces BLIMP-1, which constrains Treg proliferation and enhances suppressive functions like IL-10 secretion. It also increases chromatin accessibility at STAT5- and BLIMP-1–dependent regions associated with *CEACAM1*, leading to its upregulation and consequent dampening of TCR signaling. Moreover, *CEACAM1*^KO^ Tregs showed an increased *PRDM1* mRNA, suggesting CEACAM1 may help limit BLIMP-1. Although we have not specifically engaged CEACAM1 signaling, a major ligand for CEACAM1 is homotypic interaction with itself (9), which likely occurs in IL-2–expanded Treg cultures.

The physiological relevance of this regulatory loop remains to be determined. One possibility is that it promotes a sequential but regulated Treg response, first to autoantigen followed by IL-2–dependent amplification of these autoantigen-stimulated Tregs. This likely occurs locally, where IL-2 produced by autoantigen-reactive T cells expands relevant Tregs that in turn suppress them (53). Once suppression is achieved, IL-2 declines, and autoantigen activation and persistence of these Tregs are no longer required. As such, IL-2–dependent upregulation of BLIMP-1 and CEACAM1 may reflect a key homeostatic mechanism to ensure these Tregs are not overrepresented. Accompanying this, BLIMP-1 also enhances Treg suppressive activity, further facilitating regulation of autoreactive T cells. This mechanism may also operate during low-dose IL-2 therapy, as CEACAM1 and BLIMP-1 were upregulated in many Tregs after a 5-day induction course. Alternatively, in an environment of excess antigen and IL-2, e.g., during a robust pathogen-driven immune response, this feedback loop may restrain Tregs to allow effective immunity. Last, in therapeutic contexts where Tregs are expanded or engineered in vitro (54, 55), IL-2 exposure may promote CEACAM1 expression, potentially dampening their suppressive activity, as observed in an xGVHD model using wild-type Tregs.

## Methods

### Sex as a biological variable

PBMCs were obtained from de-identified blood donors. Although female donors were not excluded, most were male. Female recipient mice were used exclusively in xGVHD studies for consistency. Patient PBMCs from the low-dose IL-2 trial contained both sexes. Sex was not considered as a biological variable in this study.

### Study subjects

#### Human samples.

Peripheral blood samples from healthy adult donors were purchased from the Continental Blood Bank, Miami, Florida, USA. For BLIMP-1 studies with humanized mice, cryopreserved blood from healthy donors mobilized with Neupogen was obtained from the Sylvester Comprehensive Cancer Center at the University of Miami. Frozen peripheral blood samples were analyzed from 27 autoimmune patients undergoing low-dose IL-2 therapy (trial registration number, NCT01988506). Inclusion required a documented diagnosis of at least 1 of the selected diseases with mild to moderate activity and stable therapy for ≥2 months. Exclusion criteria included severe or progressive comorbid autoimmune/inflammatory disease, hematological disorders, vital organ failure, cancer, and active HIV, HBV, or EBV infections. All patients received 1 MIU/day of IL-2 on days 1-5 (induction), then every 2 weeks from day 15-180 (maintenance), with follow-up on day 240.

#### Mice.

C57BL/6J Foxp3-mRFP mice (56) and the NOD.Cg-*Prkdc^scid^* *Il2rg^tm1Wjl^*/SzJ (NSG) mice (strain 005557, The Jackson Laboratory) were maintained in pathogen-free conditions at the University of Miami animal facilities. All experiments were carried out with female mice between 8 to 12 weeks of age.

### Antibodies and flow cytometry

All antibodies are listed in Supplemental Table 1. Cell surface staining was performed with antibodies in FACS buffer (HBSS, 0.2% BSA, 0.1% sodium azide) for 15 min at 4°C. Intracellular staining was performed after fixing and permeabilizing using Foxp3/Transcription Factor Staining Buffer Set (eBioscience) according to the manufacturer’s instructions. FACS analysis was performed using BD LSRFortessa or CytoFLEX LX (Beckman Coulter), acquiring 100,000 events for PBMCs and 10,000-25,000 events for in vitro expanded Treg or Teff cells. Data was analyzed using BD FACSDiva 8.0.1 or FlowJo v10.7.1 software. Viable cells were gated based on forward versus side scatter, with doublets excluded by forward scatter area versus scatter width.

### Cell purification and culture

Human CD4^+^ T cells from heparinized leukocyte units were enriched from PBMCs that were harvested as previously described (29). Cells were stained and sorted into Tregs (CD4^+^ CD25^hi^ CD127^lo^) and T_EM_ (CD4^+^ CD25^med^ CD127^hi^ CD45RA^-^) using a BD FACS Aria-II sorter. For Treg or T_EM_ priming, sorted Treg or T_EM_ cells (5-8 x 10^5^ /well/mL) were cultured in 24 well flat-bottom plates with anti-CD3/CD28 Dynabeads (4:1 bead-to-cell ratio, ThermoFisher) and human IL-2 (500 unit/mL, Novartis) in OpTmizer CTS^TM^ T cell expansion medium (designated as SFM) (Life Technologies). When the cell density exceeded 1 x 10^6^ /mL (typically day 3 after priming), they were sub-cultured at 1-3x10^5^ /mL in fresh SFM plus human IL-2 (500 unit/mL). For CRISPR/Cas9 editing, the cells were harvested on day 3, washed once in PBS, and placed in electroporation cuvettes (5 x 10^5^/cuvette) with guide RNAs and Cas9 using the Amaxa P3 Primary Cell Kit (see below). Cells were electroporated by 4D-Nucleofecter (Lonza; program EH-115). After electroporation, 80 μl of pre-warmed SFM was added to each cuvette for 15 min at 37°C, and then the cells were replated at 5 x 10^5^ cells/mL and sub-cultured in SFM supplemented with IL-2 for downstream assays. Variations from these conditions are mentioned in the Figure legends.

To study CEACAM1 expression in vitro by FACS, total CD4^+^ T cells (5 x 10^5^) in 48 well plates (Figure 4, B and C) were cultured for 2 days, or sorted Treg/T_EM_ cells (1-2 x 10^5^) in 96 well plates (Figure 4F) were cultured for 5-6 days with soluble anti-CD3/CD28 (2 μg/mL each), human IL-2 (100 U/mL or as indicated), and/or anti-IL-2 (10 μg/mL).

### [3H]-thymidine proliferations assay

On day 7 post-transfection, cells were washed 3 times with PBS after Dynabeads removal. Cells (5-10 x 10^4^ cell/well) were cultured in SFM for 3-4 days in 96-well plates with plate-bound anti-CD3 (2 μg/mL) (OKT3, Biolegend), soluble anti-CD28 (2 μg/mL) (CD28.2, Biolegend) and human IL-2 (500 U/mL or as indicated) in 200 μL SFM. 1 μCi [^3^H]-thymidine was added 4 hours before harvest. DNA was collected using a FilterMate Harvester (Perkin Elmer), and radioactivity was measured with a MicroBetaTriLux beta scintillation counter (Perkin Elmer). Data of each sample is reported as the means of triplicate values with <10% variability.

### In vitro suppression assay

Human PBMCs (1 x 10^6^) were suspended in 1 mL SFM and labeled with 0.5 μM CFSE or Celltrace Violet (ThermoFisher Scientific) following manufacturer’s instructions. Five days post-transfection, Tregs were collected and washed 3 times with PBS after Dynabeads removal. Labeled PBMCs (1 x 10^5^ /well) were co-cultured with serial dilutions of Tregs in 48-well plates and stimulated with soluble anti-CD3 (1 μg/mL, OKT3, Biolegend) and anti-CD28 (2 μg/mL, CD28.2, Biolegend). Cultures were maintained at 37°C 5% CO_2_ for 3 days. Proliferation of CD8^+^ cells (responders) was assessed by CFSE/CellTrace dilution via flow cytometry. Suppression was calculated as: % Inhibition = [1- (%dilution of responders with Tregs/% dilution of responders only)] x 100.

### RNA isolation and RNA-Seq

RNA was isolated using the RNeasy Micro Kit (Qiagen). RNA purity, library preparation, and RNA sequencing were carried out by Novogene Corporation Inc (Sacramento, CA). RNA quality and quantity were assessed by NanoPhotometer spectrophotometer (IMPLEN, CA) and Bioanalyzer 2100 system (Agilent Technologies, CA) with RNA Nano 6000 Assay Kit, and libraries were generated using NEBNext Ultra^TM^ RNA Library Prep Kit for Illumina (NEB). Sequencing was performed with NovaSeq 6000 (Illumina, USA). Then 50 bp reads were aligned to Hg19 with TopHat2 and assembled with Cufflinks, and gene-level counts were compiled with htseq-count. Low-abundance genes were filtered using htsfilter, retaining 10,000-12,000 genes. Normalization and DEG analysis were performed with edgeR (quasi- likelihood F test). Transcripts per million (TPM) were compiled with edgeR, and an offset value of 1 was added to all TPM. Genes with TPM ≤ 2 in any genotype/condition or classified as micro/sno/sca-RNAs were removed.

Gene enrichment was performed using clusterProfiler (HGT, GSEA) with KEGG, GO, Reactome, MSigDB, or custom gene sets. GSEA rankings were based on Log2FC. GSEA plots were rendered with Enrichplot and all other plots with ggplot2 or Datagraph (Visual Data Tools Inc., USA). Additional enrichment analyses used gProfiler or Metascape.

### ATAC-Seq

50,000 unfixed nuclei from Treg or activated T_EM_ cells (3 biological replicates) were tagged using the Tn5 transposase (Nextera DNA sample prep kit; Illumina) for 30 min at 37 °C. Libraries were generated using Ad1_noMX and Ad2.1-24 barcoded primers (57) and amplified for 10-12 cycles. Fragments were purified (DNA Clean & Concentrator-5 kit, Zymo Research) and size-selected (AMPure XP, Beckman Coulter) to exclude fragments >1 kb and primers. Library quality was assessed with Agilent Bioanalyzer High-Sensitivity DNA kit. Sequencing was performed using the Illumina NextSeq 500 High Output Kit (75 bp paired-end, ≥40 million reads/sample) at the Oncogenomics Core of the University of Miami.

50 bp reads were aligned to Hg19 with bowtie2 and non-redundant reads assembled into ‘peaks’ using Genrich. These peaks, which represent ‘accessible’ genomic regions, were then annotated using Homer. Peak variance versus baseline (day 0) and differentially accessible regions (DARs) were determined using Diffbind. DNA motifs were identified and counted using SEA from MEME suite 5.4.1. DARs were cross-referenced with RNA-Seq data using standard R based operations. Genome tracks were visualized with IGV. Shown is 1 of 3 biological replicates per time point.

### Cas9 RNP assembly

The following chemically synthesized crRNAs (Integrated DNA Technologies, Newark, NJ) were used as follows: scramble control (5’-GGTTCTTGACTACCGTAATT-3’);*PRDM1* exon 2 (5’-CATTGTGAACGACCACCCCT-3’) and exon 5 (5’-CGGATGGGGTAAACGACCCG-3’); *CEACAM1* exon 2 (5’-GATGGCAACCGTCAAATTGT-3’) and exon 4 (5’- CACGCCAATAACTCAGTCAC-3’); *CEACAM1* chromatin accessibility-region 3 (5’-TCCTTTACAATCCTGTTCTG-3’ and 5’-CAGAAGGGAAATGATCTGAG-3’), region 4 (5’-GGGTTTAGAAACTGCTAGGG-3’, 5’-ATAGGGGATGAGTTAGACAC-3’, and 5’-CAGAGAGTATCTTATCTGTG-3’), and region 5 (5’-GTCATATTATTACCAGAATG-3’ and 5’-CGCCCAAACCAAGATACAGA-3’). The tracrRNA and crRNAs were reconstituted in Nuclease-Free IDTE Buffer (Integrated DNA Technologies) to 160 μM. Cas9 RNPs were prepared immediately before each experiment. crRNA and tracrRNA were mixed at 1:1 ratio, heated at 95°C for 5 min, and then cooled to room temperature on the benchtop to generate 80 μM sgRNA. 40 μM purified *S*. *pyogenes* Cas9-NLS (Macrolab, University of California, Berkeley) was slowly added to the 80 uM sgRNA at a 1:3 ratio and incubated at 37°C for 15 mins to generate Cas9 RNPs.

### T7 Endonuclease I (T7EI) assay

7 days after gene editing, cells were lysed in QuickExtract DNA Extraction Solution (ThermoFisher Scientific). Genomic DNA was PCR-amplified using the following primers: for exon 2 of *PRDM1*: forward: 5’-GCACTGTGAGGTTTCAGGGA-3’ reverse: 5’-ACCCTATGCTGCAAGTTGCT-3’; for exon 5 of *PRDM1*: forward: 5’-ATGAACTCTGCCCAAAGAATGT-3’ reverse: 5’-AGTGATGTACGTGGGTCTCTCG-3’; for exon 2 of *CEACAM1*: forward: 5’-CCTCACTTCTAACCTTCTGGTTC -3’ reverse: 5’-GGTATACATGGAACTGTCCAG -3’; for exon 4 of *CEACAM1*: forward: 5’-ATGGCCCGGACACCCCCAC -3’ reverse: 5’-CAGTGACTATGATCGTCTTGAC -3’. For *CEACAM1* chromatin accessibility regions: region 3 were forward: 5’-GGCAACATAGTGAGATCCTGTC-3’ reverse: 5’-ACAAAGCACCTGTATTCATGTTCT-3’; region 4 were forward: 5’-GGCTCCGGATATGAAAATGGATC-3’ reverse: 5’-CCTAGCCTAAGACTATGCCCATA-3’; region 5 were forward: 5’-CTCCGTCTTTCCAGTTCAAATGA-3’ reverse: 5’-GAATATCATTGCCCCTAGTCAGC-3’. PCR products were hybridized in a thermocycler with the following settings: 95°C, 10 min, 95-85°C at -2°C/s, 85°C for 1 min, 85-25°C at -0.3°C/s, 25°C for 1 min, and hold at 4°C. Reannealed DNAs were digested with T7 endonuclease I (NEB) at 37°C for 3 hours and resolved on 2% agarose gel after adding 6 x gel loading dye. The image was developed using Odyssey Fc Dual Mode Imaging System (Li-COR, Lincoln, NE).

### GVHD model and Treg transplantation

NSG mice were irradiated (2.0 Gy) 1 day before transplantation. Human PBMCs were isolated from cryopreserved mobilized blood or fresh blood of healthy donors. PBMC numbers were adjusted so that each recipient mouse received 2 x 10^6^ of T cells based on expression of CD4 and CD8. Where indicated, recipients also received in vitro expanded scrambled or *PRDM1*^KO^/*CEACAM1*^KO^ Tregs (4 x 10^6^) derived from PBMCs from the same donor. Cells were adoptively transferred in 0.2 mL of PBS i.v. through the lateral tail vein. Mice were monitored 3 times/week for body weight and GVHD clinical score (0-3 scale: hunched back, diarrhea, fur texture, and alopecia). Heparinized blood was collected after transplantation to assess Treg numbers and marker expressions by flow cytometry. Survival endpoint was defined as ≥30% weight loss. The mice transplanted with *CEACAM1*^KO^ Tregs and the relevant controls were sacrificed on day 36, and spleens were collected for flow cytometry.

### pSTAT5 and pS6 phospho-flow assay

After removal of residual Dynabeads, cells were washed 3 times with PBS and ‘rested’ in SFM (1-5 x 10^5^/mL) for 4 hours. Cells were then stimulated with IL-2 for 15 min at 37 ^°^C. pSTAT5 staining was performed as previously described (29). For pS6 analysis, cells were ‘rested’ overnight in SFM and stimulated as indicated (Figure 7F) with plate bound anti-CD3 (OKT3, Biolegend), soluble anti-CD28 (CD28.2, Biolegend), and human IL-2 (100 unit/mL) for 6 hours at 37°C. After stimulation, cells were fixed, permeabilized, and stained for pS6 staining as described for pSTAT5.

### Reverse transcription polymerase chain reaction (RT-PCR)

Complementary DNA (cDNA) samples were synthesized from purified RNA using the High-Capacity cDNA Reverse Transcription Kit and oligo (dT) primers (ThermoFisher Scientific). Primers for target sites ranging from exon 2 to exon 5 of *PRDM1* are forward: 5’-GCACTGTGAGGTTTCAGGGA-3’, reverse: 5’-AGTGATGTACGTGGGTCTCTCG-3’. Primers used for determining *CEACAM1* isoforms are forward: 5’ GCTCTACCACAAGAAAATGG, reverse: 5’ CATTGGAGTGGTCCTGAG. PCR conditions were: 95°C 5 min (1 cycle), 95°C 30s, 59°C 1min, 72°C 1min, (40 cycles), 72°C 10 min (1 cycle), and hold at 4°C.

### Western blotting

Cell extracts were prepared using cell extraction buffer (ThermoFisher Scientific, Rockford, IL) with protease inhibitors and 1 mM PMSF (Sigma-Aldrich). Equal amounts of protein (200 μg/lane) were resolved on 10% SDS-PAGE gels and transferred to membranes. Blots were probed with anti-BLIMP-1 (6D3, eBiosciences), anti-CEACAM1 (283324, R&D Systems), anti-β-tubulin (Cell Signaling), and anti-β-actin (Biolegend) antibodies. Detection was performed using ECL (ThermoFisher) and imaged using Odyssey Fc Dual Mode Imaging System (Li-COR, Lincoln, NE).

### Luciferase assay

Regions of open chromatin within the CEACAM1 locus (region 3: chr19: 42501707-42502106; region 4: chr19: 42498277-42498676; region 5: chr19: 42487142-42487541) were cloned into the pGL4.12[luc2CP] luciferase reporter vector (Promega, WI) using XhoI and BglII restriction sites. To generate mutated constructs, sequences corresponding to STAT or PRDM1 binding motifs were deleted as follows: STAT motifs on region 3 (AAAAAAACAAAACAAAAAG; ACTTCCTCAGA; and AGTTTCCAGAAA); STAT motifs on region 5 (TTCCAGGAAA and TTTCTTGGAA); and *PRDM1* motifs on region 4 (TGCTTTTCTCT and GAGAGGGAGAGGAA). IL-2–responsive HEK-Blue IL-2 cells were purchased from InvivoGen. To assess BLIMP-1–dependent transcription, cells were cotransfected with 0.25 μg of empty vector (pcDNA3.1(+)-P2A-eGFP, GenScript, NJ) or a BLIMP-1 expression plasmid (PRDM1_OHu19113C_pcDNA3.1(+)-P2A-eGFP, GenScript, NJ), along with 0.13 μg of pGL4.12[luc2CP]-based luciferase reporter constructs containing wild-type or *PRDM1*-mutated CEACAM1 regions. For STAT-dependent transcription, cells were transfected with wild-type or *STAT-*mutated reporter constructs. Transfections were performed using GeneJuice transfection reagent (Novagen) following the manufacturer’s protocol. After transfection, cells were treated with or without IL-2 (500 U/ml) for 24 hours. Luciferase activity was measured using the Promega Luciferase Assay System and quantified on a BioTek Synergy LX Multi-Mode Reader.

### Statistics

Data was analyzed using GraphPad Prism 8. Results are shown as means ± SEM. Statistical significance was assessed using paired or unpaired 2-sided *t* test, 1-sample 2-sided *t* test, or 1-way and 2-way ANOVA with multiple comparisons as indicated in figure legends. For survival analyses, a log-rank (Mantel-Cox) test was performed. For 1-sample *t* tests, values were normalized to a control set at 1. Significance was defined as *P <* 0.05.

### Study approval

Cryopreserved blood obtained from the Sylvester Comprehensive Cancer Center was approved by Institutional Review Board of the University of Miami (protocol #20160363). The clinical trial and collection of blood samples were approved by the French regulatory authority and Ethical Committee (EudraCT: 2013-001232-22). All patients gave written informed consent. All animal procedures were approved by the Institutional Animal Care and Use Committee (IACUC, protocol #23-152) of the University of Miami.

### Data availability

Gene Expression Omnibus: RNA-Seq data have been deposited under accession codes GSE221348 and GSE259322. Numerical data is available in a XLS file.

## Author contributions

Conception and design were done by TRM, AY, YD, RBL, and DK. Acquisition of data was done by YD, AY, MV, SNC, and AM. Analysis and interpretation of data were done by YD, AY, LN, MD, NT, MR, SNC, AVV, RBL, and TRM. Manuscript writing was done by TRM, YD, and AY. All authors edited and approved the manuscript.

## Supplementary Material

Supplemental data

Unedited blot and gel images

Supporting data values

## Version 1 08/07/2025

In-Press Preview

## Version 2 09/23/2025

Electronic publication
