# Supplementary material for: BLIMP-1 and CEACAM1 cooperatively regulate human Treg homeostasis and function to control xenogeneic GVHD
Source: JCI Insight. 2025 Aug 7;10(18):e183676. doi: 10.1172/jci.insight.183676 (PMC12487853; doi:10.1172/jci.insight.183676)
Supplement: Supplemental data [file jciinsight-10-183676-s023.pdf]

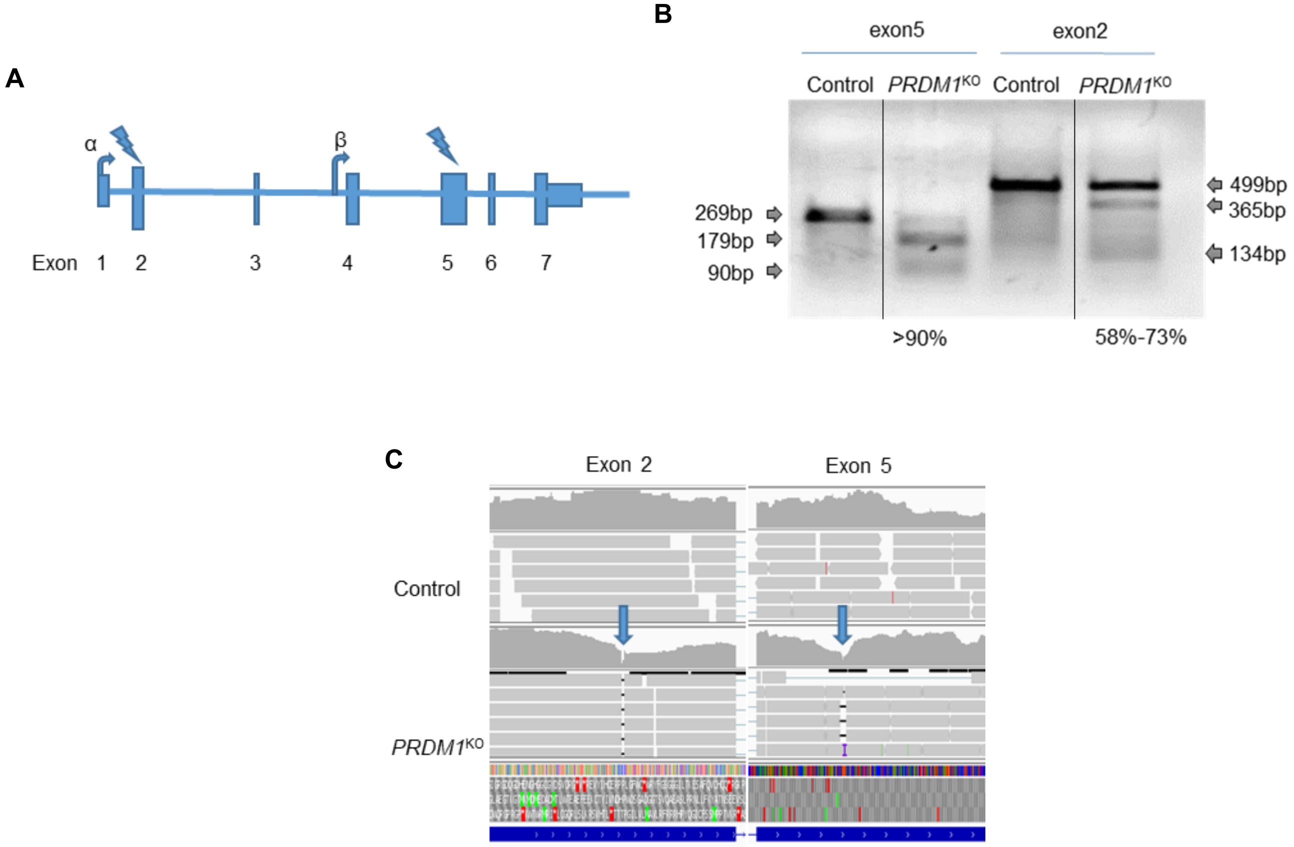

Supplemental Figure 1. Efficient knockout of *PRDM1* in expanded human Tregs using CRISPR-Cas9. (**A**) Dual-target knockout strategy on *PRDM1*. Two sgRNAs targeting exon 2 and exon 5 were designed to increase knockout efficiency. (**B**) T7 endonuclease I (T7EI) assay demonstrates genome editing in both exon 2 and exon 5 of *PRDM1* loci. Expected PCR product size (499bp and 269bp, respectively) and approximate expected sizes of T7EI-digested fragments are indicated. Quantifications of knockout efficiency of three independent experiments are shown. The black vertical lines indicate that lanes were run on the same gel but were noncontiguous.(**C**) Integrative genomics viewer tracks of RNA-sequencing data show the decreased depth of reads and indels on the target sites after gene editing. The dash line indicates deletion and I indicates insertion.

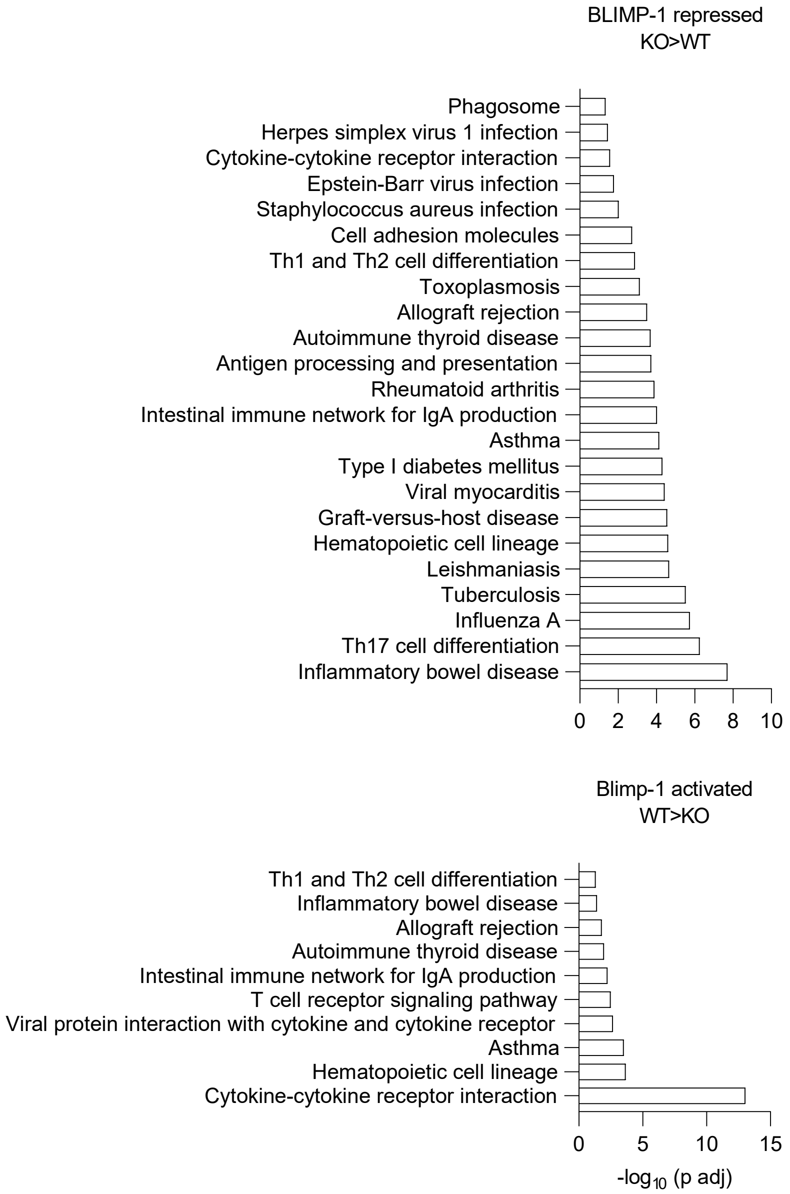

Supplemental Figure 2. 7 days after transfection, control and *PRDM1*^{KO} Tregs were cultured in media overnight and then stimulated with anti-CD3/CD28 and IL-2 for 16 hr for RNA-seq analyses. KEGG pathway analysis of DEGs.

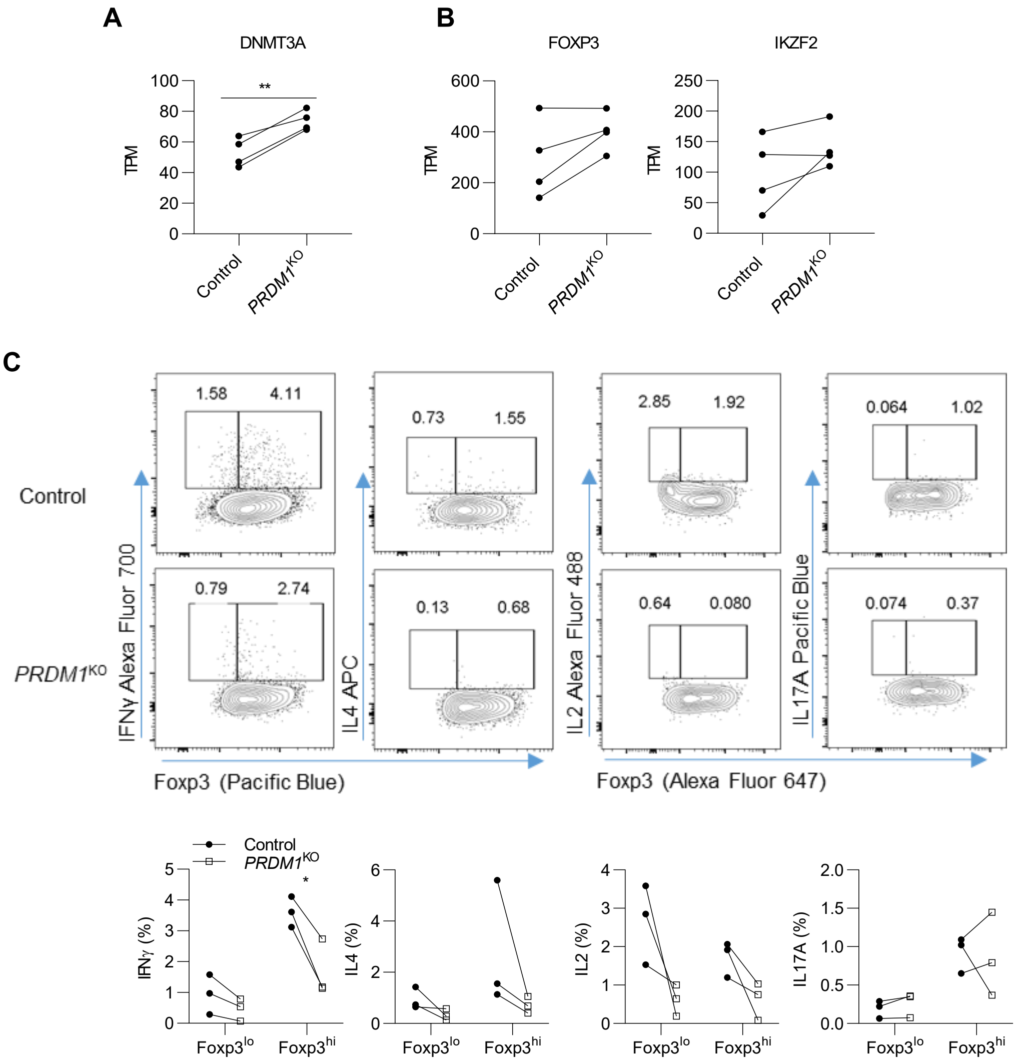

Supplemental Figure 3. (**A-B**) RNA expression of (**A**) *DNMT3A* or (**B**) *FOXP3* and *IKZF2* by RNA-seq in control and *PRDM1*^{KO} Tregs (n=4) (**C**) Flow cytometric analysis of indicated cytokine production in control and *PRDM1*^{KO} cells (gated on Foxp3^{lo} and Foxp3^{hi}) after stimulation with anti-CD3/CD28 and IL2 (Representative, top; and quantification, bottom, n=3). Data are shown as the mean±SEM and were analyzed by a paired two-sided *t* test, $^{*}p < 0.05$, $^{**}p < 0.01$

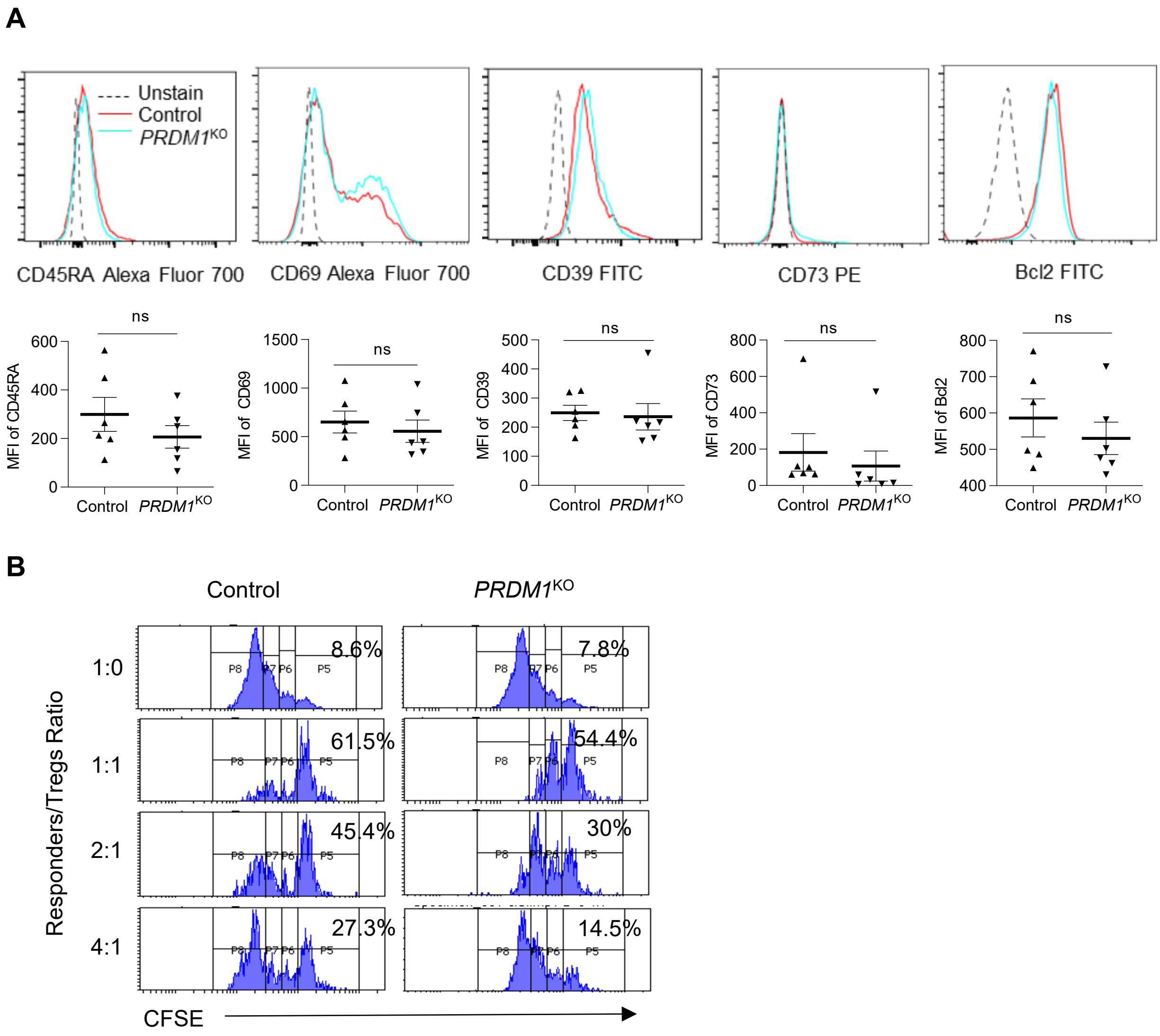

Supplemental Figure 4. (**A**) Expression levels of proteins associated with Treg activation and suppression. Representative histograms (top) and quantification (bottom, n=6) of indicated markers in control and *PRDM1*^{KO} Tregs. Data are shown as the mean ±SEM and were analyzed by a paired two-sided *t* test, ns, not significant. (**B**) Representative histograms of *in-vitro* suppression assay shown on Figure 3E. Proliferation of responder cells (gated CD8^{+} T cells) were traced using CFSE. Percentage of non-proliferative cells were indicated.

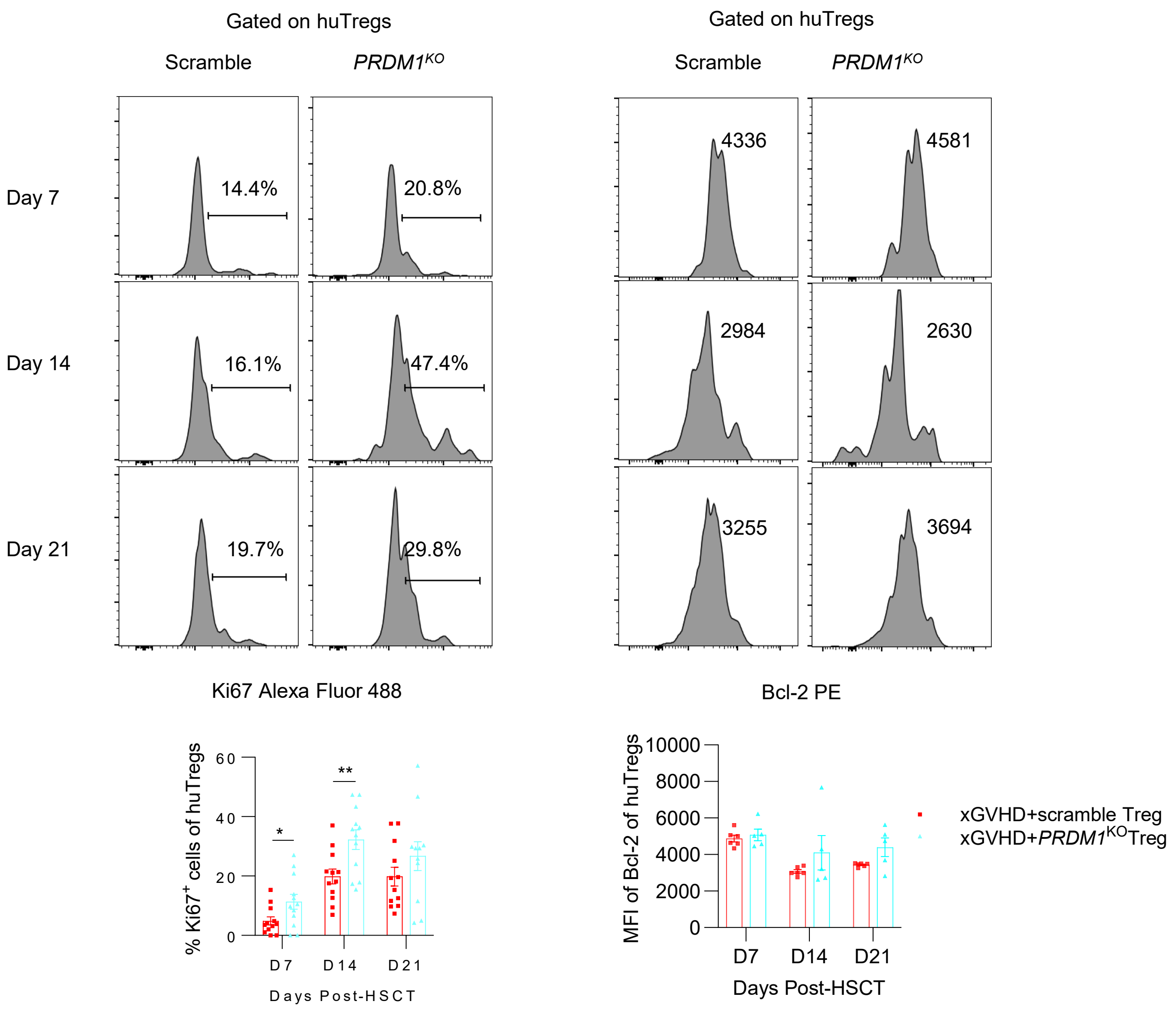

Supplemental Figure 5. Human PBMCs together with scramble or *PRDM1*^{KO} human Tregs were transplanted into irradiated NSG mice to assess the suppressive activity of Tregs on xeno GVHD. Mice were bled on day 7, 14 and 21 post-transplant. Expression of Ki67 (left; n=12) and Bcl-2 (right; n=6) on huTregs in the blood were determined by flow cytometry. Data were analyzed by a multiple unpaired two-sided t-test, $^{*}p < 0.05$, $^{**}p < 0.01$.

Cell sorting strategy

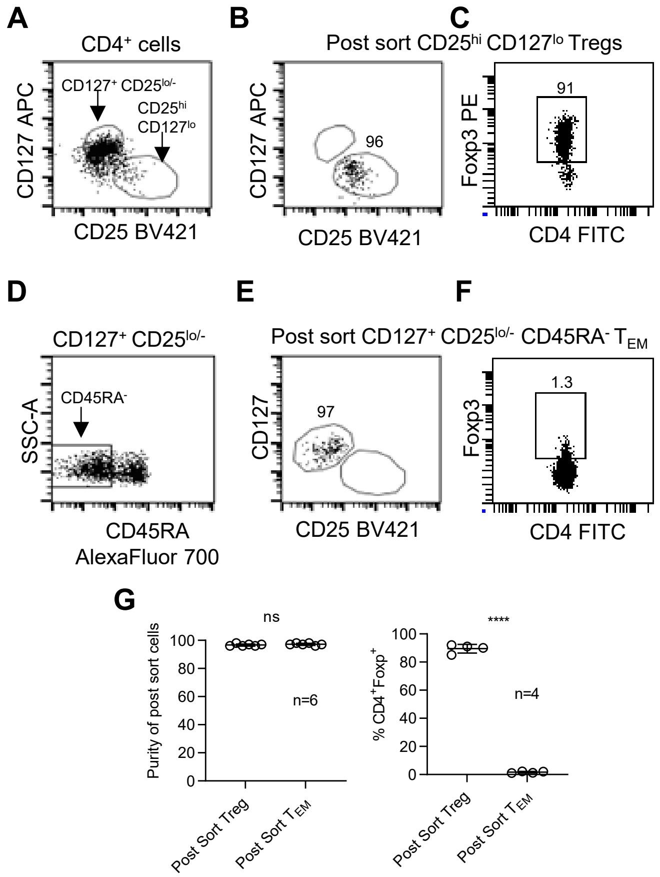

Supplemental Figure 6. Cell sorting strategy to isolate CD4$^+$ Treg and T_{EM} cells. Peripheral blood CD4$^+$ T cells were enriched using anti-CD4$^+$ magnetic beads. CD4$^+$ Tregs were obtained by sorting (**A**) CD4$^+$ CD25^{hi} CD127^{lo} cells [≥96%$^+$ post sort, (**B**)], which were typically >90% Foxp3$^+$ (**C**). CD4$^+$ T_{EM} cells were obtained by sorting CD4$^+$ CD127^{hi} CD25$^{lo/-}$ cells (A) that were also CD45RA- (**D**) [≥97%$^+$ post sort, (**E**)], which were 1-2% Foxp3$^+$ (**F**). Quantitative data for each sample is shown in (**G**). Data were analyzed by two-sided t-test. ****$p<0.0001$; ns, not significant.

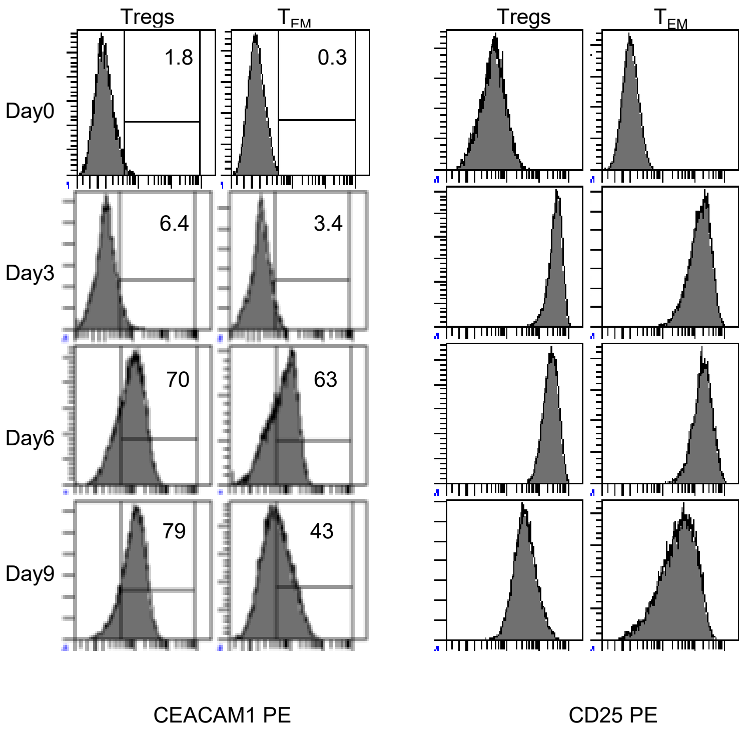

Supplemental Figure 7. Human $CD4^+$ $CD25^{hi}$ $CD127^{lo}$ Tregs or $CD4^+$ $CD45RA^-$ $CD25^{med}$ $CD127^{hi}$ T_{EM} cells were sorted from purified $CD4^+$ T cells from healthy donors. The purified cells were stimulated at culture initiation with anti-CD3, anti-CD28, and IL-2 and sub-cultured with IL-2 on days 3 and 6. Representative CEACAM1 (left) and CD25 (right) expression was determined by flow cytometry. Note: CEACAM1 and CD25 were identified in separate tubes with a distinctive flow panel.

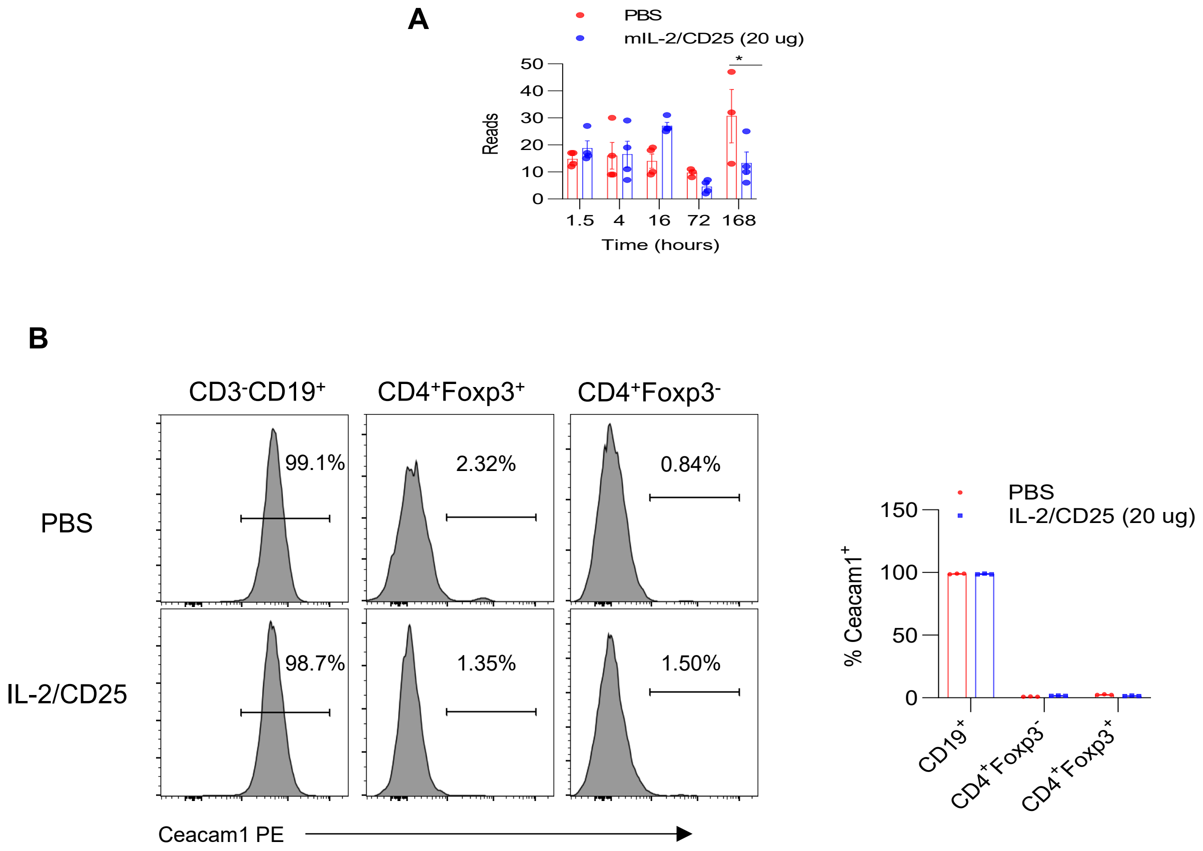

Supplemental Figure 8. Expression of Ceacam1 on mouse cells. (**A**) C57BL/6J-FOXP3-mRFP mice were injected with PBS or 20 μg/kg mIL-2/CD25 fusion protein and spleen were collected at the indicated time points. $CD4^+$ Tregs were sorted from the splenocytes for RNAseq analysis (n=4). RNA data were expressed as read counts. Data were analyzed by two-way ANOVA with multiple comparisons. $^*p < 0.05$. (**B**) Mice were treated with PBS or mIL-2/CD25 fusion protein (20 μg/kg) twice per week for 2 weeks. Ceacam1 expression on the indicated cells from spleen was determined by flow cytometry. Representative histograms (left) and quantitative data (right, n=3).

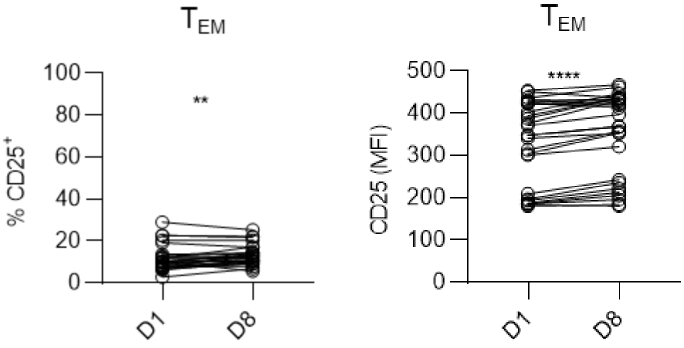

Supplemental Figure 9. Quantitative data of CD25 expression on $CD4^{+}T_{EM}$ from all patients. Data were analyzed by unpaired two-sided t-test. **p<0.01, ****p<0.0001.

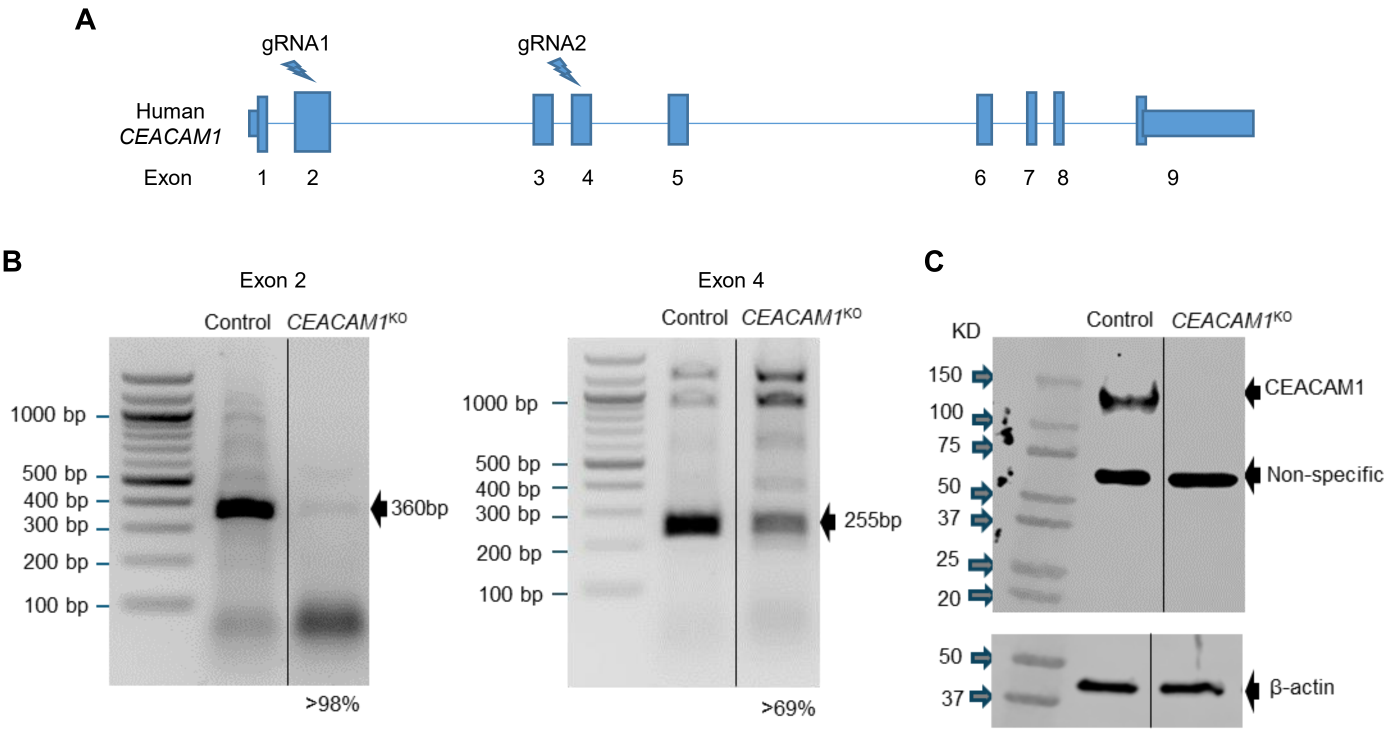

Supplemental Figure 10. Knockout of *CEACAM1* in human Tregs using CRISPR-Cas9. (**A**) Dual-target knockout strategy for *CEACAM1*. Two sgRNAs targeting exon 2 and exon 4 were designed to increase knockout efficiency. (**B**) T7 endonuclease I (T7EI) assay of gene editing in exon 2 and exon 4 of *CEACAM1*. Expected PCR product size and knockout efficiency are indicated. (**C**) CEACAM1 expression was determined by immunoblotting in scramble control and *CEACAM1*^{KO} Tregs. The black vertical lines on (**B**) and (**C**) indicate that lanes were run on the same gel or blot but were noncontiguous.

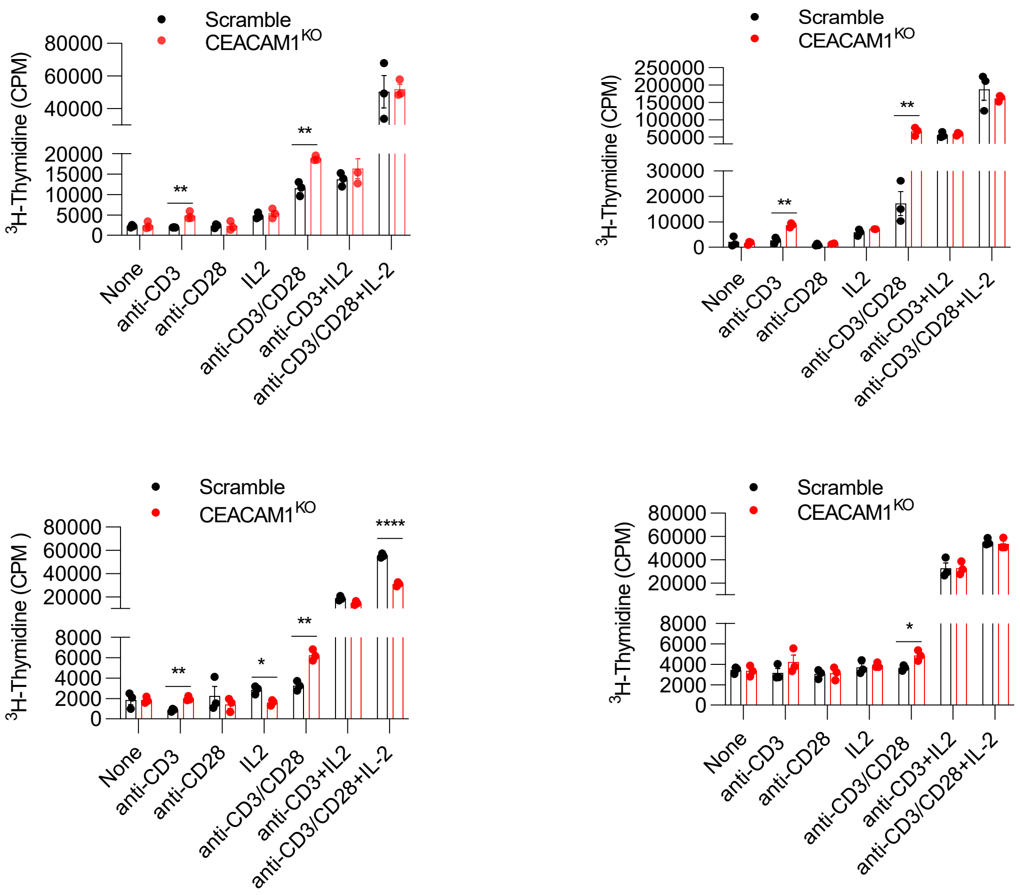

Supplemental Figure 11. Biological replicates of proliferation assay assessed by ^{3}H-thymidine incorporation. Data with each experiment were analyzed by multiple unpaired t-test (mean ± SEM in each experiments show technical replicates). These data were used to determine the fold-change for each of the four biological replicates on Figure 7C. *p<0.05, **p<0.01, ****p<0.0001.

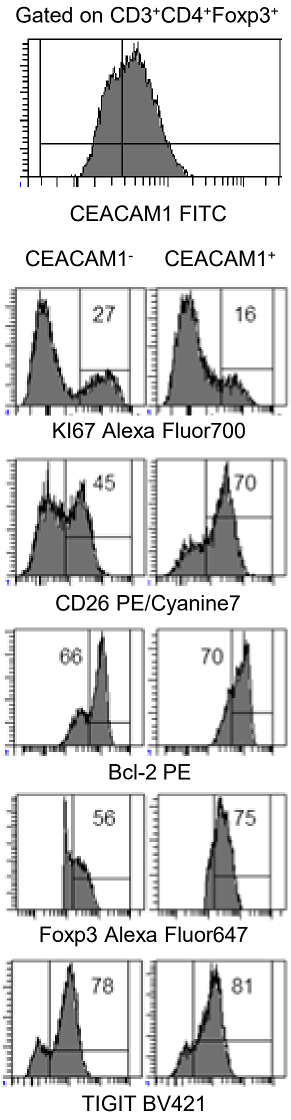

Supplemental Figure 12. Representative histograms of the indicated markers for CEACAM1+ and CEACAM1- Tregs on D8 after low-dose IL-2 therapy.

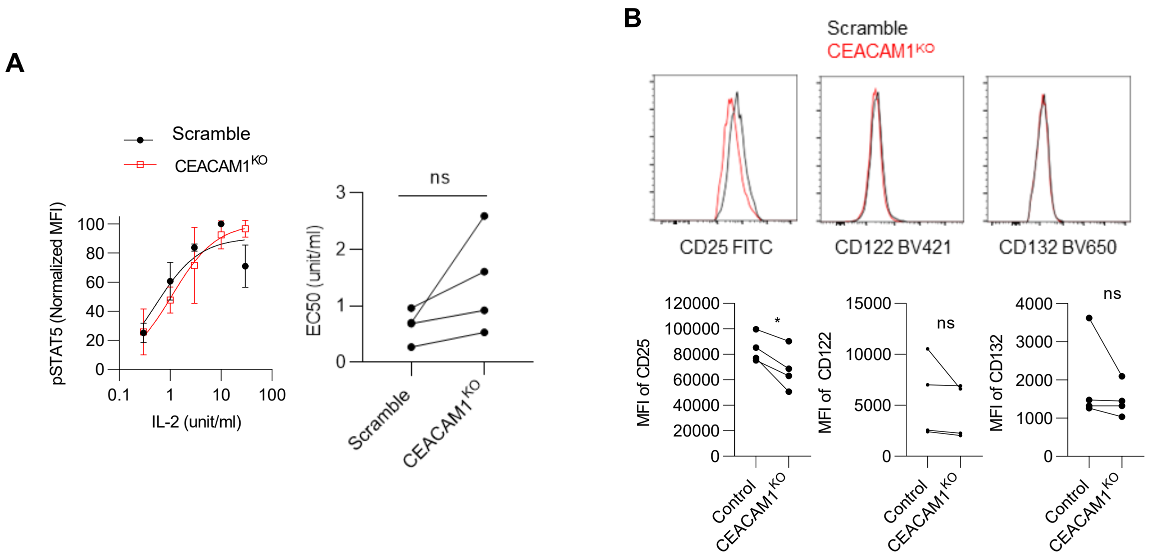

Supplemental Figure 13. CEACAM1 expressing Tregs are associated with largely normal IL-2R signaling. (**A**) After the 7-day expansion culture with IL-2, Scramble and *CEACAM1*^{KO} Tregs were rested overnight and then treated with IL-2 for 15 minutes. Nonlinear regression analysis of IL-2-induced pSTAT5 (left; n=4; mean ± SEM) and quantitative data of EC50 (right; n=4). (**B**) Expressions of IL-2R subunits were determined by flow cytometry; representative histograms (top) and quantitative data (bottom; n=4). Data were analyzed by a paired two-sided t-test. *p<0.05; ***p<0.001; ****p<0.0001, ns, not significant.

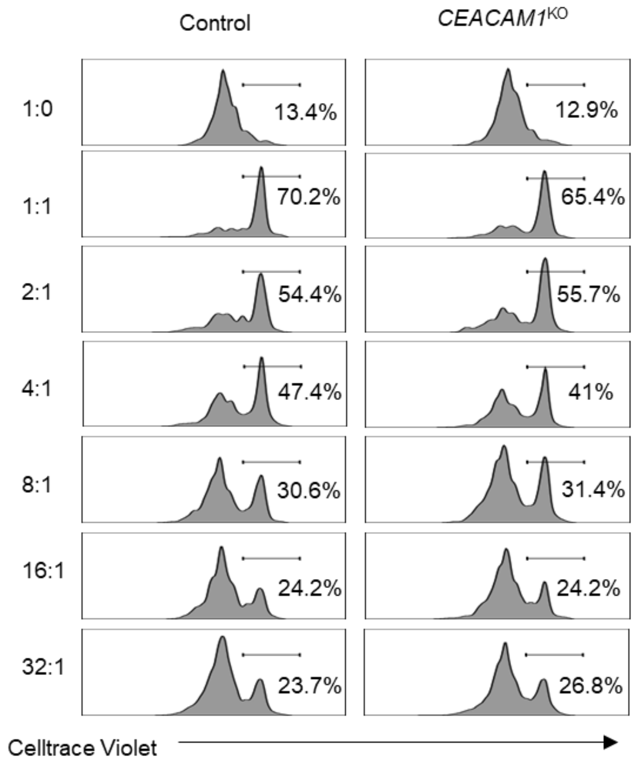

Supplemental Figure 14. Representative histograms of celltrace violet traced proliferation of responder cells (gated on $CD8^+$ T cells) in the *in vitro* suppression assay shown in Figure 8A.

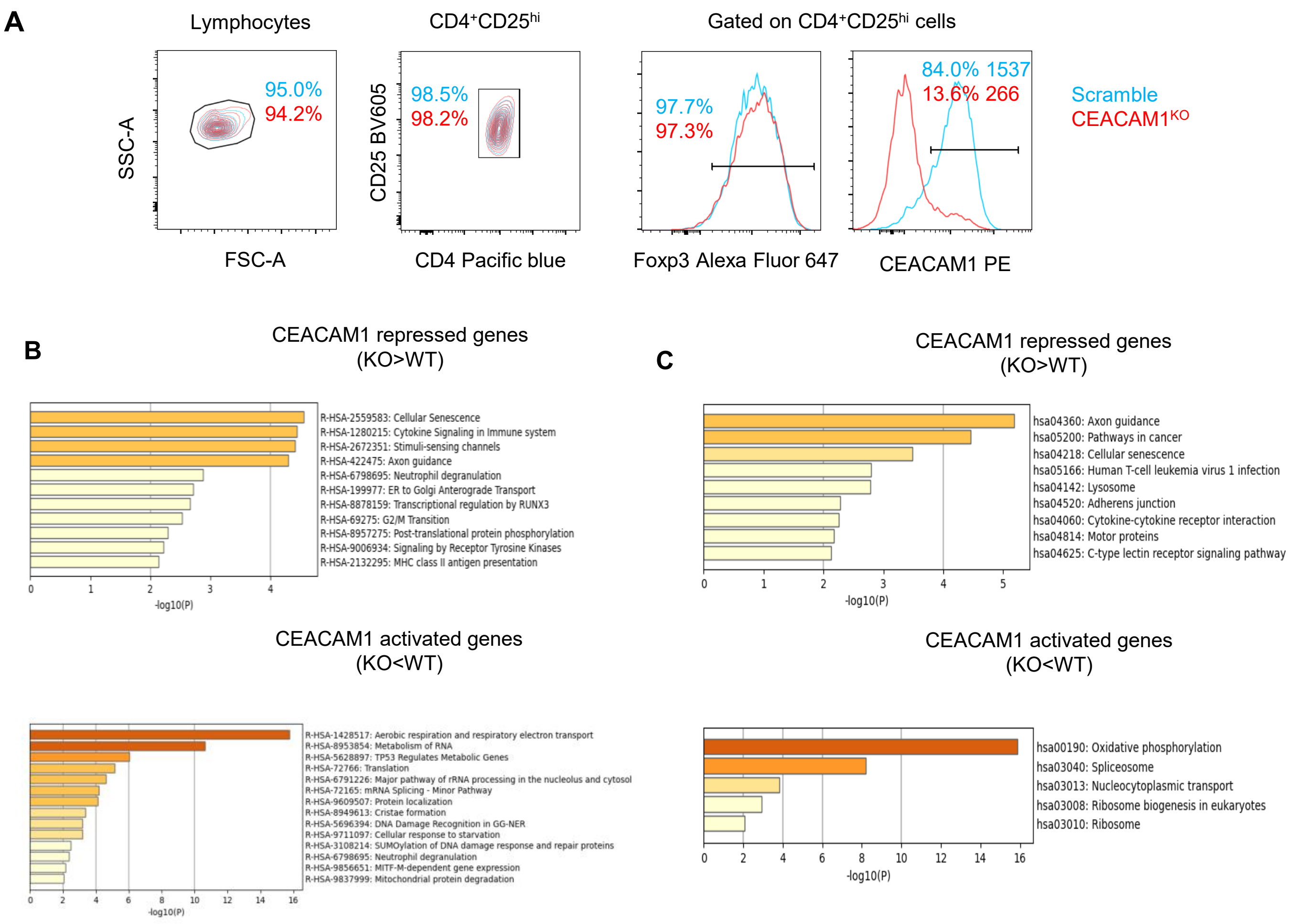

Supplemental Figure 15. (**A**) Expression of Foxp3 and CEACAM1 on expanded scramble and *CEACAM1*^{KO} Tregs were examined by flow cytometry before transplantation on day 0. Percentage of Foxp3^{+} cells was indicated. CEACAM1 expression was shown by both percentage of CEACAM1^{+} cells and total MFI of CEACAM1 on Tregs. (**B-C**) RNA-seq analysis was performed using input control and *CEACAM1*^{KO} human Tregs before transplantation. (**B**) Reactome pathway analysis of DEGs. (**C**) KEGG pathway analysis of DEGs.

Supplemental Table 1: List of antibodies

Manufacturer	Antibody name	Clone	Catalog number
Monoclonal anti-human antibodies			
Biolegend (San Diego, CA)	FITC-αCD4	RPA-T4	300506
	PerCP-Cy5.5-αCD4	RPA-T4	300530
	Pacific Blue-αCD4	RPA-T4	300521
	APC-αCD127	A019D5	351316
	APC-Cy7-αCD45RA	HI100	304128
	Alexa Fluor 700-αCD45RA	HI100	304120
	BV711-αCD3	OKT3	317328
	PE-αCD25	M-A251	356104
	BV421-αCD25	M-A251	356114
	BV605-αCD25	M-A251	356142
	Alexa Fluor 488-αCD25	BC96	302616
	PerCP-Cy5.5-αCD25	BC96	302626
	BV650-αCD132	TUGh4	338614
	PE-αCTLA4	BNI3	369604
	FITC-αCD39	A1	328206
	BV605-αCD73	AD2	344024
	PE-αCD73	AD2	344004
	Alexa Fluor 700-αCD69	FN50	310922
	PE-Cy7-αCD26	BA5b	302714
	BV421-αTIGIT	A15153G	372710
	BV605-αPD1	EH12.2H7	329924
	Alexa Fluor 700-αIFNγ	B27	506516
	APC-αIL4	MP4-25D2	500812
	Alexa Fluor 488-αIL2	MQ1-17H12	500314
	Pacific Blue-αIL17A	BL168	512312
	PE-αFoxp3	259D	320208
	Alexa Fluor 647-αFoxp3	259D	320214
	Pacific Blue-αFoxp3	259D	320216
	FITC-αHelios	22F6	137214
	Alexa Fluor 488-αKi67	11F6	151204
	PE-αBcl2	100	658708
	Alexa Fluor 488-αBcl2	100	658704
	BV605-αCD19	HIB19	302244
BD Biosciences (San Jose, CA)	PerCP-Cy5.5-αCD127	HIL-7R-M21	560551
	BV421-αCD122	Mik-β3	562887
	FITC-αCD66	B1.1	551479
	BV421-αCTLA4	BNI3	562743
	Alexa Fluor 700-αKi67	B56	561277
	FITC-αphosphorylated STAT5 (pSTAT5)(pY694)	47/Stat5(pY694)	612598
	PE-αpS6 (pS235/pS236)	N7-548	560433
ThermoFisher Scientific (Vilnius, LT)	αhuman IL-2	AB12-3G4	16-7027-85
	Fixable Viability Dye eFluor 455UV		65-0868-14
R&D Systems (Minneapolis, MN)	PE-αhuCEACAM1/CD66a	283340	FAB2244P
Monoclonal anti-mouse antibodies			
BD Biosciences (San Jose, CA)	FITC-αCD45	30-F11	553080
	APC-αCD8	53-6.7	553035
Biolegend (San Diego, CA)	Pacific Blue-αCD19	6D5	115523
	BV650-αCD4	RM4-5	100555
ThermoFisher Scientific (Vilnius, LT)	PerCP-Cy5.5-αCD3e	145-2C11	45-0031-82
	PE-αmCD66a/Ceacam1	CC1	12-0661-80
