# Supplementary figures and images for: BLIMP-1 and CEACAM1 cooperatively regulate human Treg homeostasis and function to control xenogeneic GVHD
Source: JCI Insight. 2025 Aug 7;10(18):e183676. doi: 10.1172/jci.insight.183676 (PMC12487853; doi:10.1172/jci.insight.183676)

Fig.1 B

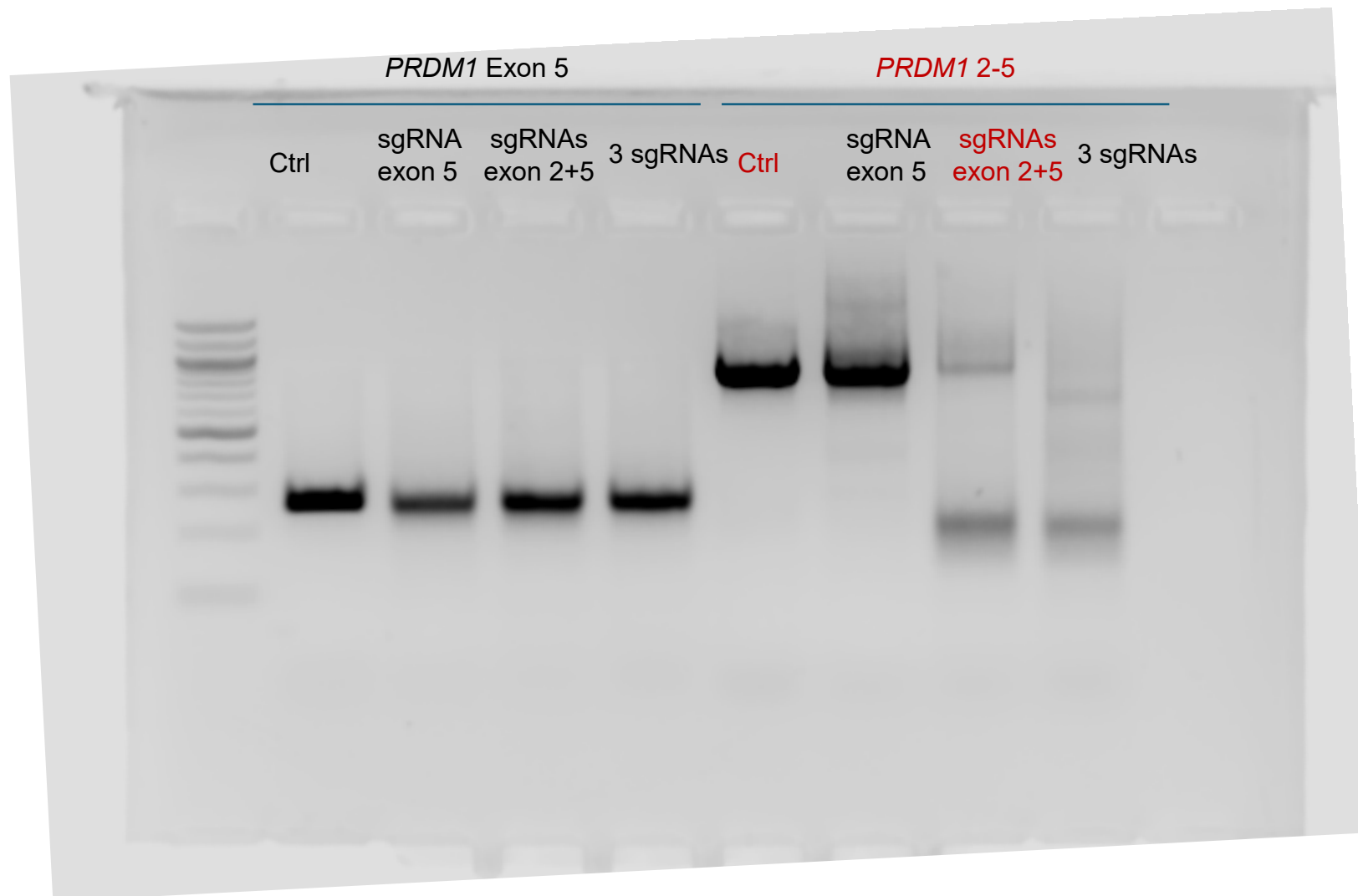

Fig.1C

BLIMP-1

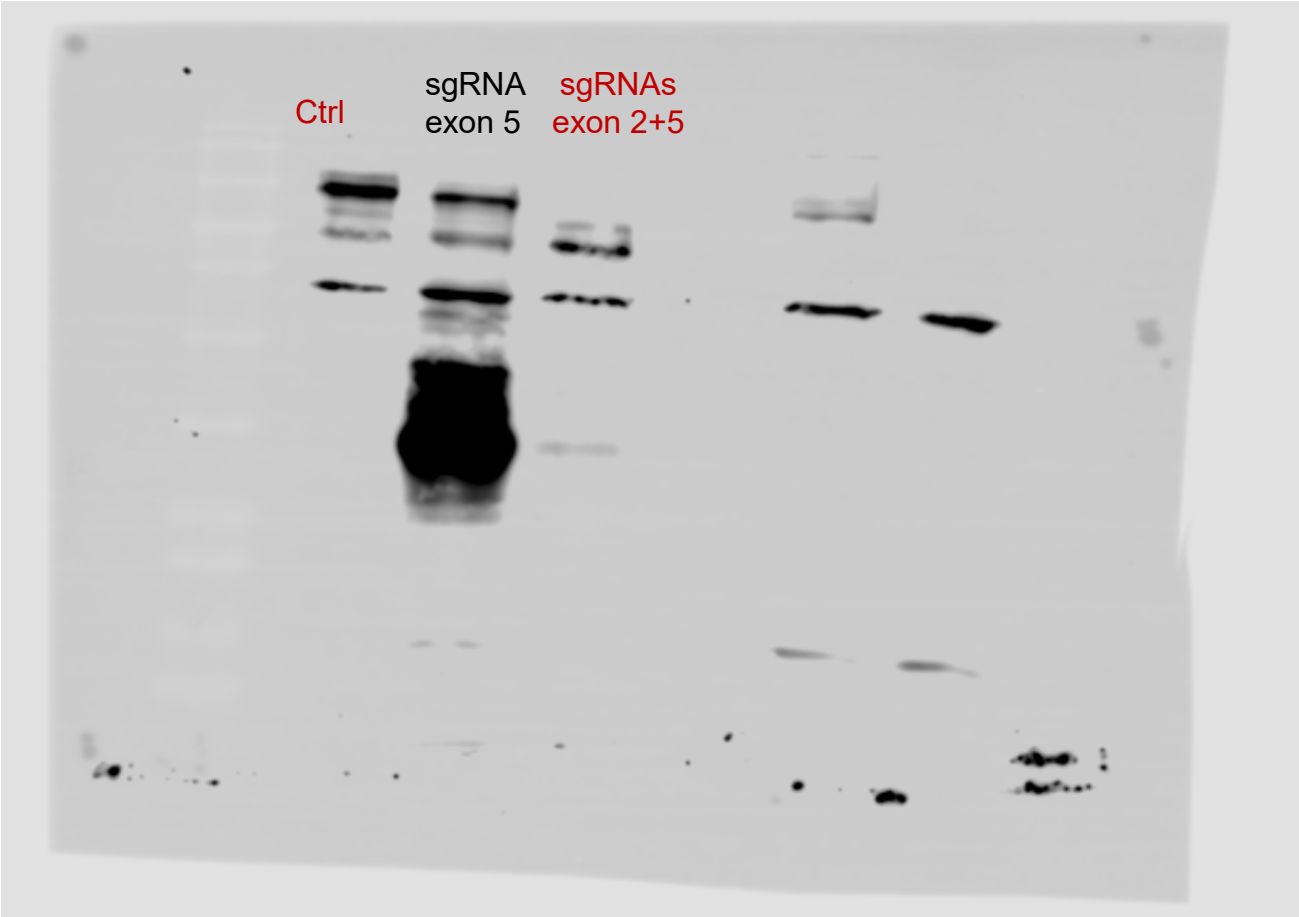

Tubulin

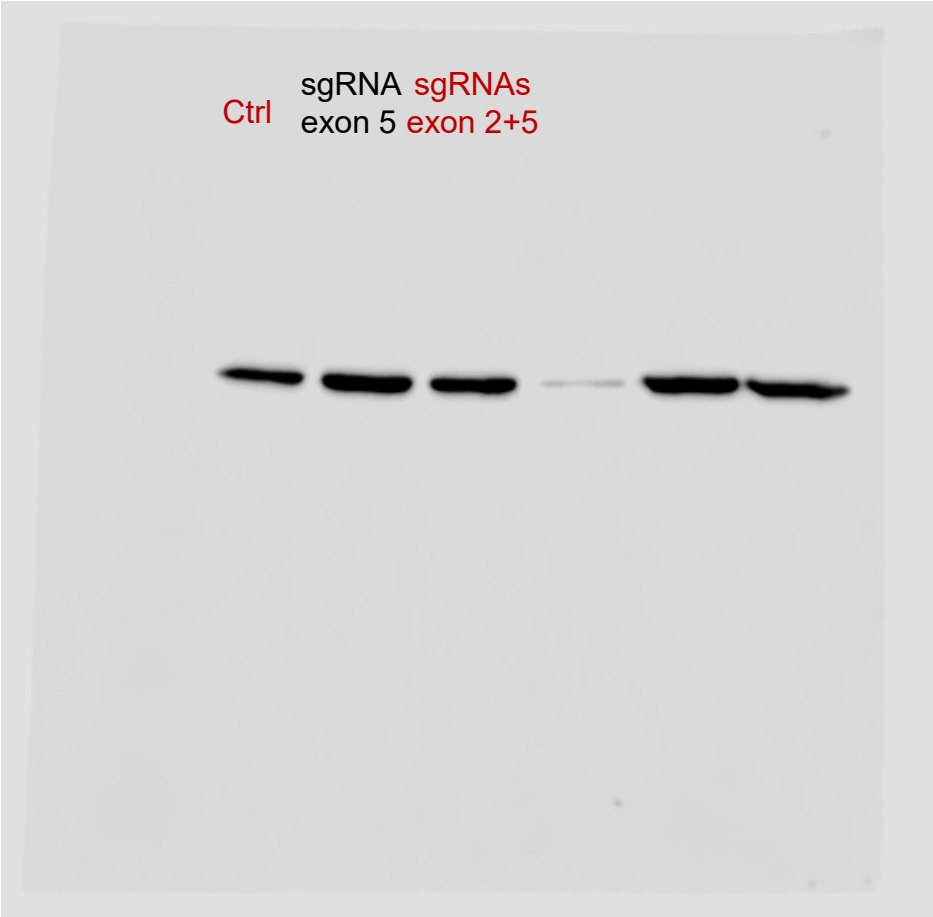

Fig.6C

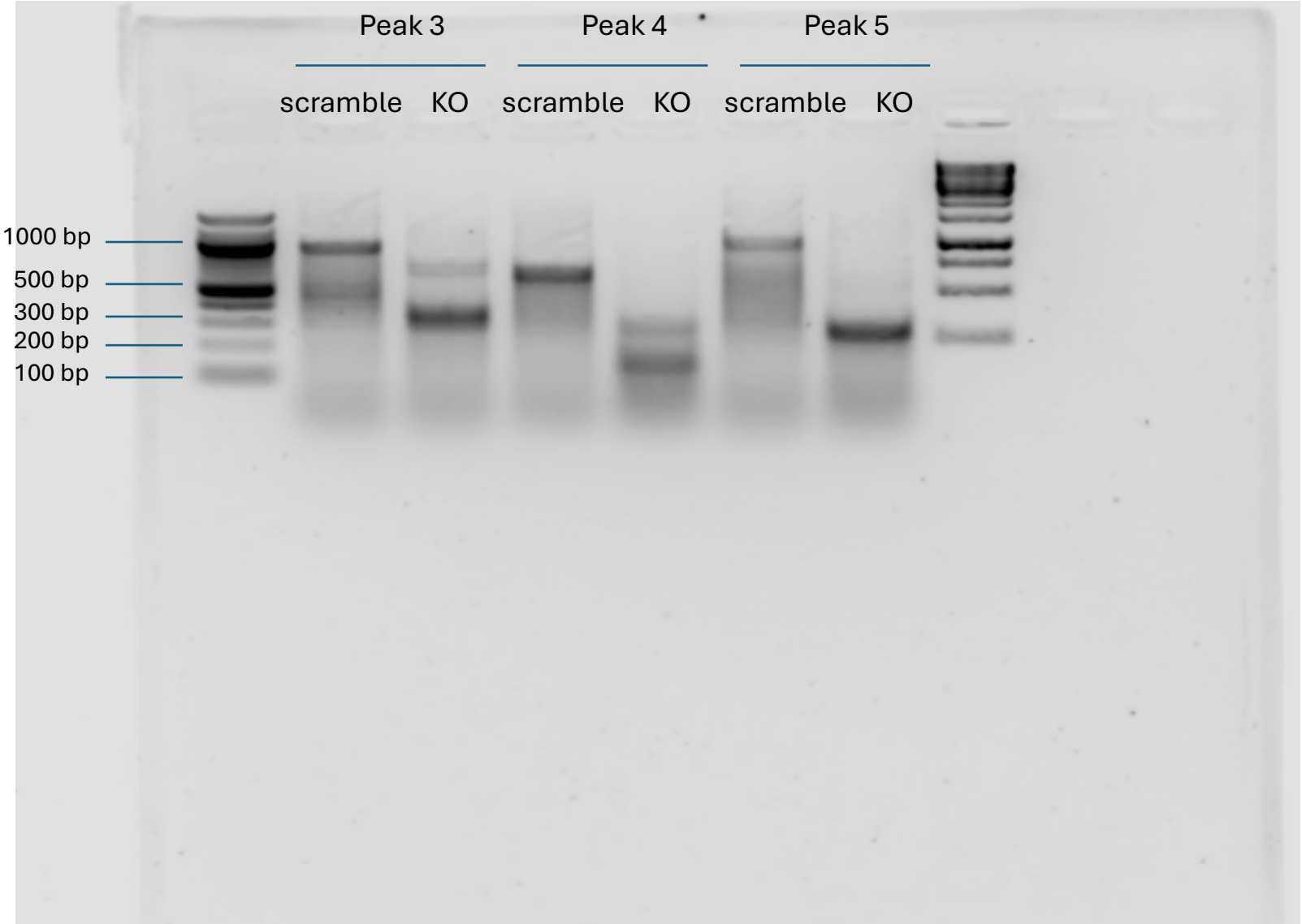

Fig.6H

BLIMP-1

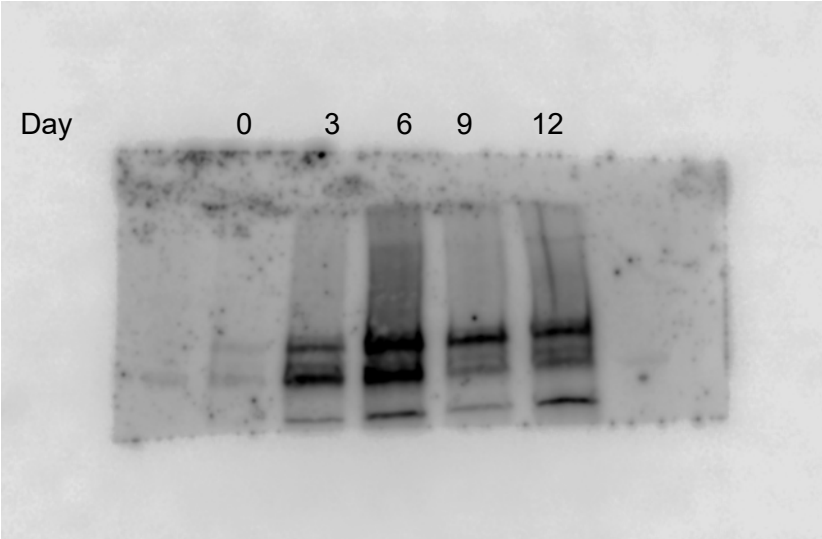

$\beta$ -actin

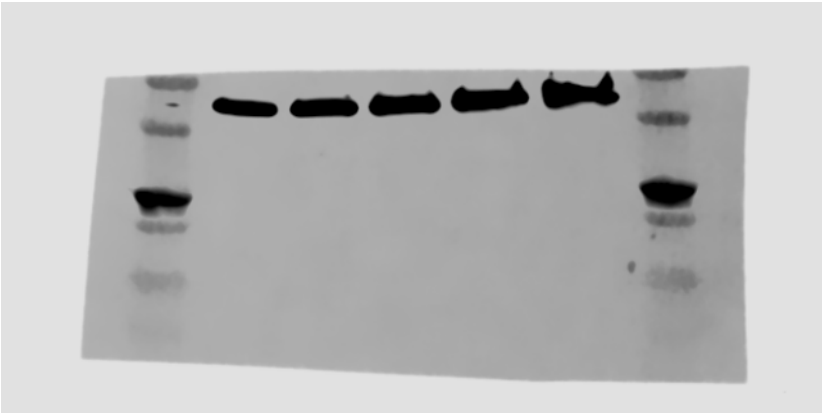

Fig.7A

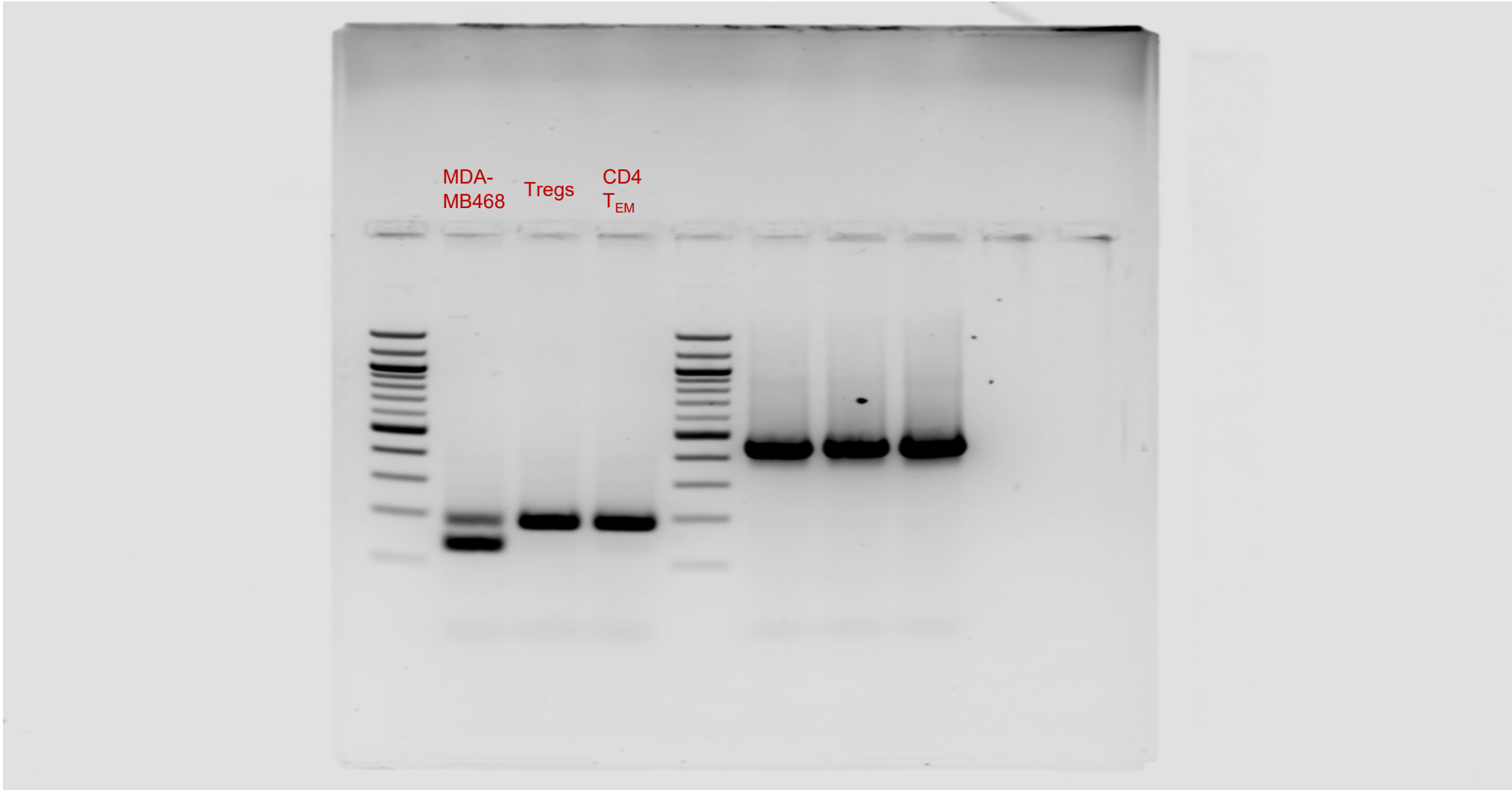

Fig. S1B

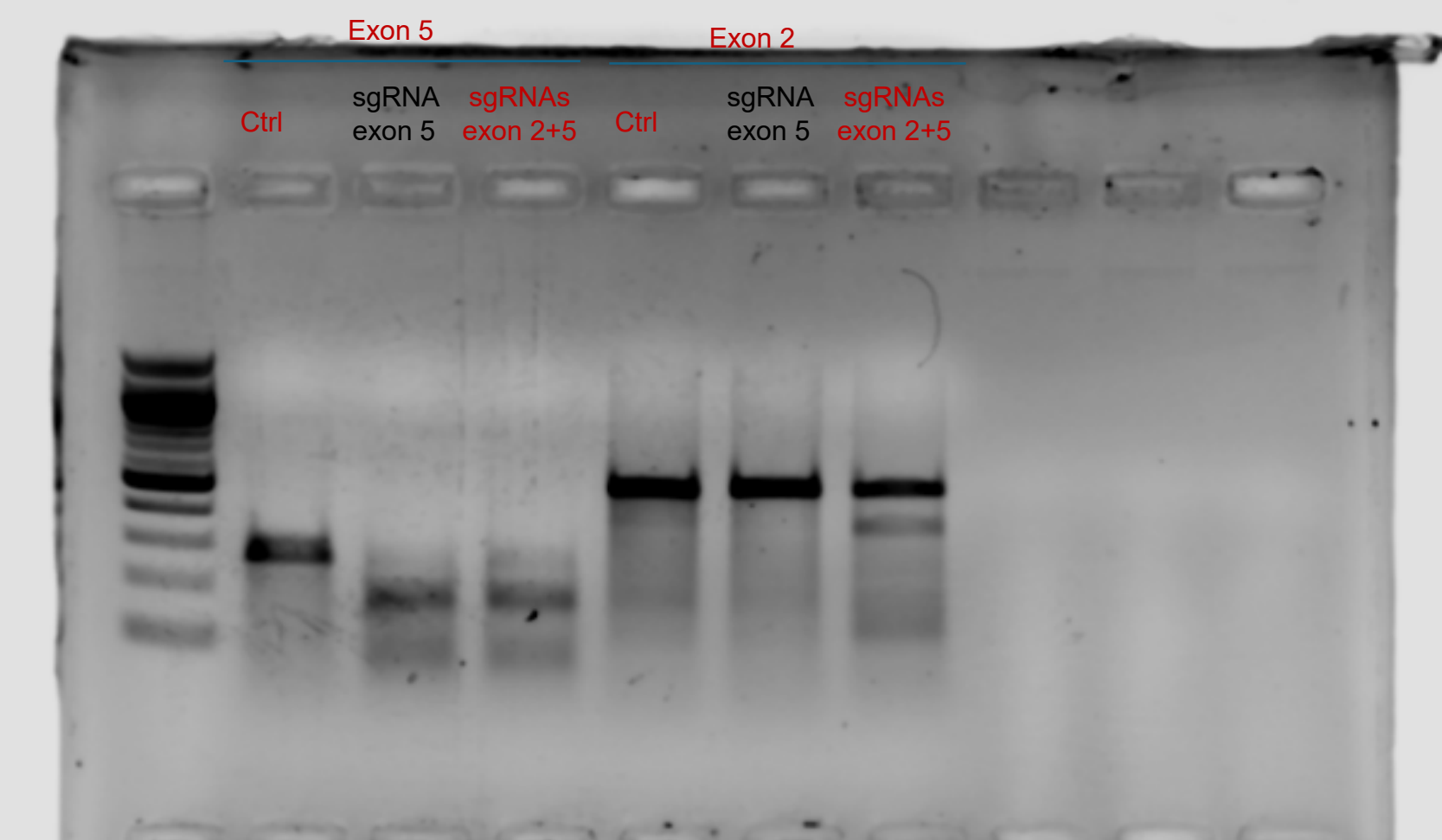

Fig. S10B

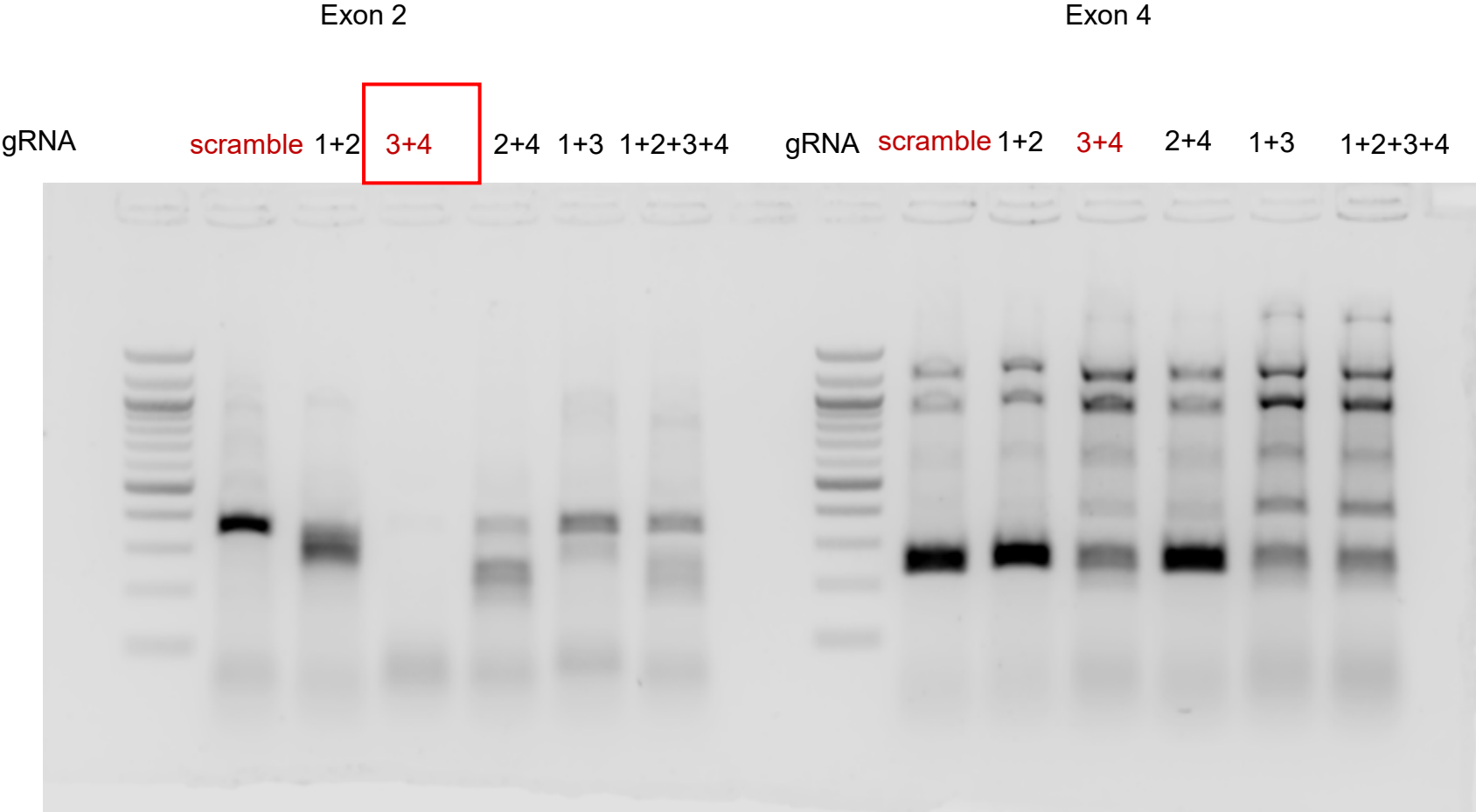

Fig. S10C

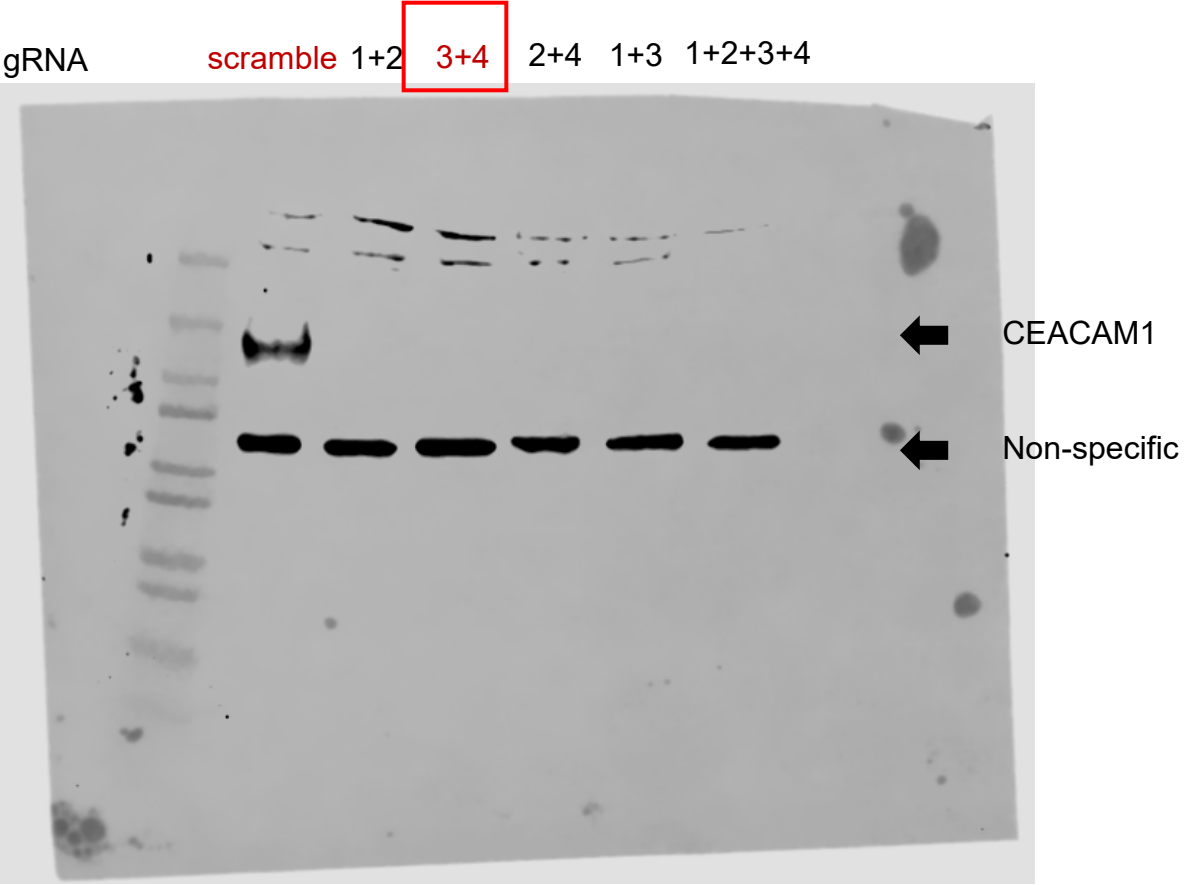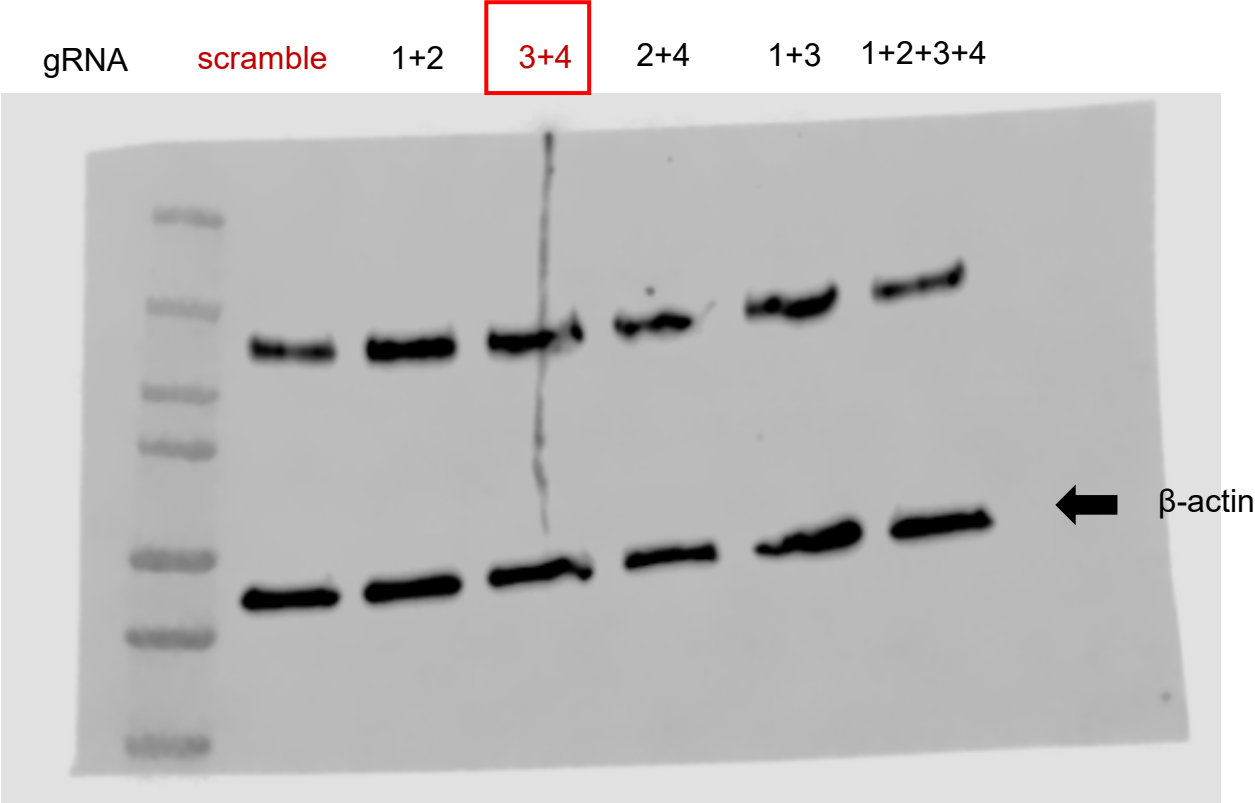

Supplement: Unedited blot and gel images [file jciinsight-10-183676-s024.pdf]
